# Genomic investigation reveals evolution and lifestyle adaptation of endophytic *Staphylococcus epidermidis*

**DOI:** 10.1038/srep19263

**Published:** 2016-01-13

**Authors:** Vasvi Chaudhry, Prabhu B. Patil

**Affiliations:** 1 CSIR- Institute of Microbial Technology, Sector 39A, Chandigarh-160036 (India)

## Abstract

*Staphylococcus epidermidis* is a major human associated bacterium and also an emerging nosocomial pathogen. There are reports of its association to rodents, sheep and plants. However, comparative and evolutionary studies of ecologically diverse strains of *S. epidermidis* are lacking. Here, we report the whole genome sequences of four *S. epidermidis* strains isolated from surface sterilized rice seeds along with genome sequence of type strain. Phylogenomic analysis of rice endophytic *S. epidermidis* (RESE) with “type strain” unequivocally established their species identity. Whole genome based tree of 93 strains of *S. epidermidis* revealed RESE as distinct sub-lineage which is more related to rodent sub-lineage than to majority of human lineage strains. Furthermore, comparative genomics revealed 20% variable gene-pool in *S. epidermidis*, suggesting that genomes of ecologically diverse strains are under flux. Interestingly, we were also able to map several genomic regions that are under flux and gave rise to RESE strains. The largest of these genomic regions encodes a cluster of genes unique to RESE that are known to be required for survival and stress tolerance, apart from those required for adaptation to plant habitat. The genomes and genes of RESE represent distinct ecological resource/sequences and provided first evolutionary insights into adaptation of *S. epidermidis* to plants.

*Staphylococcus epidermidis* is a well-known member of human micro-flora and recognized to have established commensal relationship with its host^1^. *S. epidermidis* is also known to be an emerging nosocomial pathogen and its infections estimated to be costing 2 billion dollars annually in US^2^,^3^. However, the clinical life-style is thought to be more of “accidental” side in contrast to its original symbiotic life-style^4^,^5^,^6^. Further, *S. epidermidis* is increasingly being recognized as reservoir of genes required for natural adaptation on its host tissues that are in turn acquired by other predominantly pathogenic species, like *S. aureus*^7^,^8^,^9^. Apart from human, *S. epidermidis* is a remarkably diverse species reported in a multitude of diverse environments such as fermented sausage^10^,^11^, dry-cured lacon^12^,^13^, domesticated animals^14^ as well as from wild mouse species^15^ and meat^16^.

Interestingly, there are also many reports, using both culture dependent and culture independent approaches to demonstrate the presence of strains belonging to genus *Staphylococcus* in general and *S. epidermidis* in particular as normal constituents of plant microbiome^17^,^18^,^19^,^20^,^21^. Previous studies have reported *S. epidermidis* for its plant protection, plant growth and development abilities^20^,^21^,^22^,^23^. Apart from this, presence of *S. epidermidis* as a major member of microbiome of bryophytes^24^, attest plants as natural and primitive habitats of this bacterium. Hence, there is a need to understand evolutionary relationship and differences in *S. epidermidis* isolates from plants with those isolated from animals. However, previous studies on plant associated *S. epidermidis* were based on 16S rRNA gene sequence which severely limits detailed comparative studies at inter-strain level.

On the other hand, whole genome sequence can provide finest and comprehensive insights into relationship and also difference in a bacterial group upto strain level^25^,^26^,^27^,^28^. Whole genome sequence of nearly hundred strains of *S. epidermidis* are available in public database and all these strains are from human origin, except for few from those of meat and mouse origin^15^,^16^. However, not a single genome sequence is available from non-human and non-animal origin. To the best of our knowledge there are no studies that address comparative genomics of *S. epidermidis* from diverse lifestyles. Hence there is need to understand the relationship and difference of plant associated *S. epidermidis* in greater detail using whole genome level to gain novel insights into ecology and evolution of species “*epidermidis*”.

## Results and Discussion

### Beyond 16S rRNA- Whole genome based strain typing of rice endophytic *Staphylococcus epidermidis* (RESE) isolates

In this study, we isolated 54 bacteria from surface sterilized rice seeds (see methods). Bacterial endophytes were screened morphologically and initially typed using partial 16S rRNA gene sequence analysis. Out of 54, 13 isolates (representing 24% of the endophytes) were identified as *Staphylococcus* species. Out of 13, 4 isolates belonged *S. epidermidis* which we refer below as rice endophytic *S. epidermidis* (RESE) and have selected for further analysis.

Plants are known to be the habitat of diverse species of *Staphylococcus*, including the *S. epidermidis*. In one study, a phyllosphere isolate was reported to have plant beneficial properties such as hydrolytic enzyme activity, siderophore production and volatiles synthesis for inhibiting phytopathogenic fungi *R. solani*, which in turn positively influence the development of plants^20^,^22^,^23^. Another study reported plant growth promoting traits such as siderophore production, phytohormones (IAA and GA) production^29^.

Hence, plant associated *S. epidermidis* isolates might represent ecologically and evolutionarily distinct strain or lineage of *S. epidermidis*. However, molecular typing of plant origin *S. epidermidis* isolates is restricted to sequencing the 16S rRNA, which can be near identical between novel species as in case of *S. aureus*^20^,^21^,^25^. To address this issue, we conducted whole genome sequencing and analysis of RESE to establish their identity as *S. epidermidis.* Type strains are descendants of the original isolates that were defined in species and subspecies descriptions and therefore, play a crucial role in defining the phylogenomic and taxonomic space of Bacteria^30^. Therefore, we also sequenced the genome of “type strain” of *S. epidermidis* MTCC3382(T), which was originally isolated from human nares^31^. The draft genome features and assembly stats of *S. epidermidis* MTCC3382(T) and RESE genomes are shown in Table 1 and Table S1, respectively. All RESE and MTCC3382(T) have almost similar genome size and GC content which is typical of *S. epidermidis* (Table 1). Even the number of CDS is also typical of *S. epidermidis.* This suggests that there has no drastic alteration in genome size or reductive evolution in RESE isolates.

Average Nucleotide Identity (ANI) has emerged as powerful genome based criteria for establishing species identity along with Genome-Genome Distance Calculator or digital DNA-DNA hybridization (dDDH). Therefore, to confirm the phylogenomic identity of RESE, we calculated ANI of all the RESE with MTCC3382(T). While ANI represents core genome, dDDH represent variable genome. All the four RESE isolates showed ANI of 97% which is much above the cutoff of 95% for delineating species^32^. Similarly, dDDH value was 75% with “type strain”, which is above the cut-off 70% for delineating species^33^. Both ANI and dDDH confirmed that RESE belong to *S. epidermidis* (Table 2). To our knowledge, this is the first report of whole genome sequencing of *S. epidermidis* from plants or non-human/animal origin in particular and are important resource for understanding evolution of *S. epidermidis*.

### Distinct phylogenetic and evolutionary history of RESE isolates

Although phylogenomic criterion established species status of RESE as *S. epidermidis*, ANI values of 97% also indicate that the RESE strains have diverged 3% after their origin from common ancestor. *S. epidermidis* is one of the few microbes with rich genomic data available in public domain. There are more than 100 genomes of *S. epidermidis* isolates in NCBI belonging to human, rodents and processed animal food. This gave us a wonderful opportunity to look in depth the inter-strain phylogenetic relationship.

**Figure 1:**
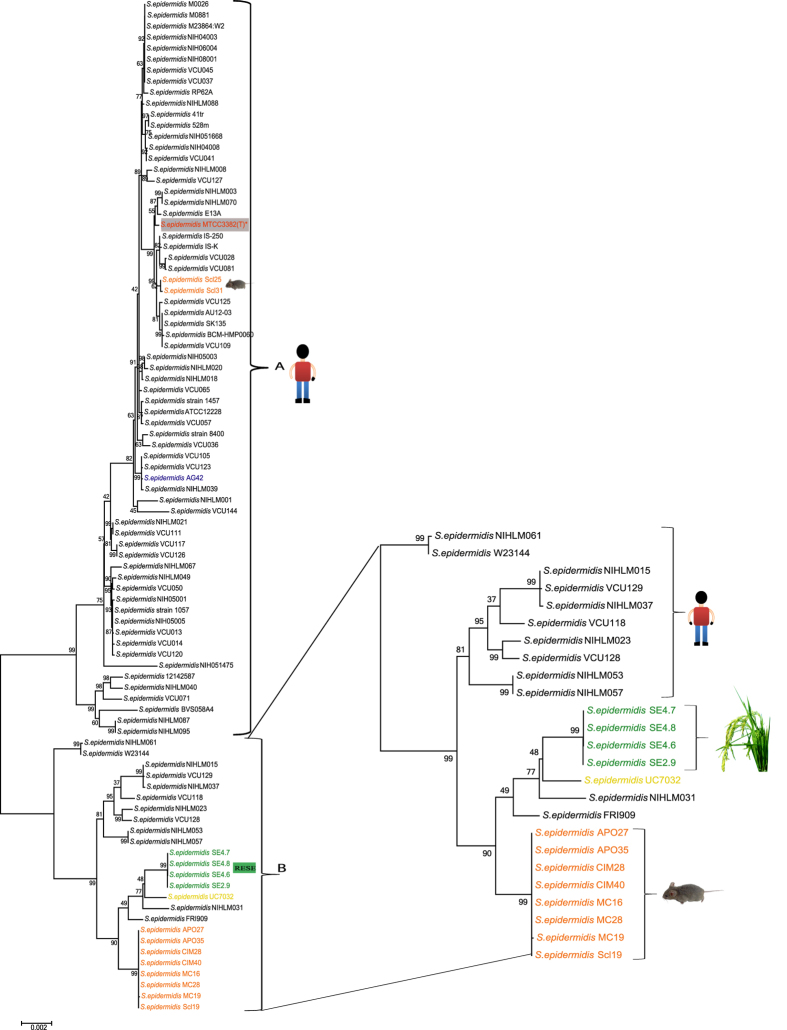
Maximum likelihood tree of different *S. epidermidis* constructed on 31 housekeeping genes using General Time reversible model (Gamma distributed with Invariant sites (G + I) method with 500 bootstrap replications two distinct groups, called A and B. “Type strain” *S. epidermidis* MTCC3382(T) under study is highlighted in grey box with red letters and asterisk mark. Strain highlighted in Blue is of Sheep rumen and Yellow is a pig meat isolate. Strains names with isolation source and accession numbers are listed in Table S2.

We carried out population-level genome based phylogenomic analysis of *S. epidermidis* isolates [88 from NCBI database, 4 RESE and MTCC3382(T)] as shown in Fig. 1, Table S2. Interestingly, all the 93 strains formed two phylogenetic distinct groups, called A and B. Among them, 64 human nosocomial and commensals, two rodents and one sheep associated *S. epidermidis* strains along with “type strain” MTCC3382(T), were mapped to the phylogenomic group A. In contrast, 8 rodents, 1 pig meat isolate and 12 human isolates along with four RESE formed another group B. Interestingly, all the RESE strains formed a separate sub-lineage, suggesting their distinct phylogenetic history (Fig. 1).

**Figure 2:**
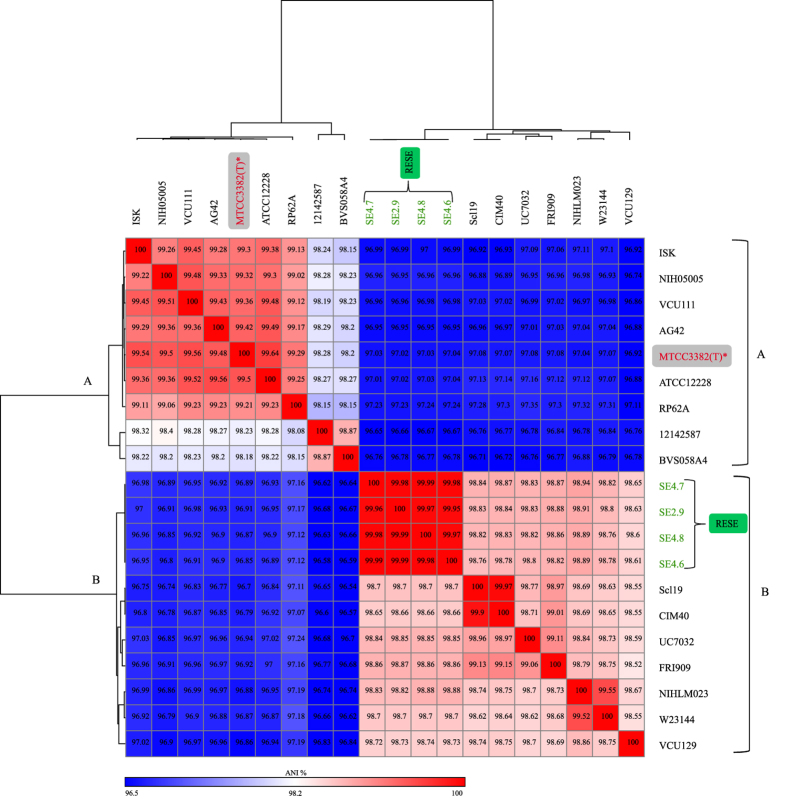
Heat-map of Average Nucleotide identity (ANI) values amongst different lineages/sub-lineages representative strains of *S. epidermidis* revealing two major groups. “Type strain” *S. epidermidis* MTCC3382(T) is highlighted in grey box with red letters and asterisk mark, RESE strains are highlighted with green letters and green box. Strains names with isolation source and accession numbers are listed in Table S2.

Further, to confirm the findings from phylogenetic analysis, we also carried out heat-map analysis based on ANI values of different lineages and sub-lineages representatives of *S. epidermidis*. The results again confirmed our above finding that there are two major groups of *S. epidermidis*, one corresponds to the majority of the human associated *S. epidermidis*, along with “type strain” MTCC3382(T) and other group consists of RESE, rodents along with a pig meat isolate. (Fig. 2). Indeed such a large scale population based phylogeny based on genomic information not only revealed different evolutionary history of majority of human isolates (including “type strain”) but also RESE and rodent strains. It is surprising that the *S. epidermidis* isolates from rodent that made their own sub-lineage are not associated with main human lineage A, but belonged to other major lineage B, that are also consisted of RESE isolates (Fig. 1). Further, RESE isolates are just 1% different at ANI from rodent isolates but 3% different at ANI from human isolates, including “type strain” (Fig. 2). Phylogenetically both rodent and RESE sub-lineages are closely related suggesting a shared phylogenetic history. Interestingly, a recent study reported the sourdough isolates of *Lactobacillus reuteri*, a well known gut commensal, was also phylogenetically closer to rodent strains than to strains of main human lineage^34^. Such a parallel phylogenetic history of two gram-positive bacteria is quite striking. This warrants further in-depth genomic studies on commonalities of rodent and plant microbiome.

At the same time, even RESE and rodent isolates differ by 1% at ANI and 10% at dDDH values, suggesting that there is also lot of genomic difference between them. As mentioned earlier, RESE strains differ by 3% at ANI, with majority of human associated *S. epidermidis* while at dDDH they differ by 25% (Table S3). It is important to note that ANI, takes into account only common regions of the genome, dDDH takes into account both common and variable genome. Hence, apart from understanding the relationship by ANI, characterizing core genome will allow us to understand the relationship while the variable part of the genome can provide us insights into origin and adaptation of these lineages to different habitats.

### Pangenomic analysis distinguishes isolates of plant and animal origin

DNA-DNA hybridization (DDH) is expected that two strains of same species can vary up to 30% in their DNA^33^. Accordingly, modern view of prokaryotic genomes distinguishes ‘core’ genome sequences, which are common to all strains of a particular species, from the ‘accessory/variable’ genome sequences, which are not universally distributed^35^. Hence, along with core genome it is also necessary to study the accessory/variable part of the genome. The amount and type of variable part may potentially determine the ability of *S. epidermidis* to be a versatile bacterium and its adaptation to plant host.

**Figure 3:**
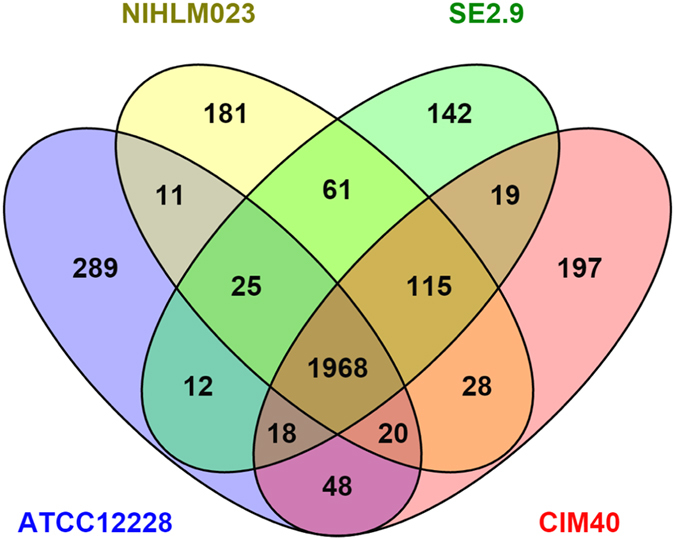
Pangenome analysis of major lineages representative of *S. epidermidis* ATCC122278 (Human), NIHLM023 (Human), SE2.9 (RESE) and CIM40 (Rodent) depicted in Venn diagram.

To gain clue into core and variable gene-pool among *S. epidermidis* isolates from diverse phylogenetic and ecological origin, we carried out pan-genomic studies. The analysis revealed a core-gene pool of nearly 2000 (n=1968) genes that is conserved and comprised of core of *S. epidermidis* and around 400 genes in each isolate that form non-core or variable gene pool (Fig. 3). Among the variable gene pool, which constitutes nearly 20% of total gene-pool, we were interested in the genes that are unique to each strain and in turn give advantage to adapt to diverse habitats. The four strains, ATCC12228, NIHLM023, SE2.9 and CIM40, which represented diverse lineages and ecology have 289, 181, 142 and 197 unique genes respectively (Fig. 3; Table S4–S7). While human strain ATCC12228 has higher number of unique genes, the RESE strain SE2.9 has relatively lesser number of unique genes.

**Figure 4:**
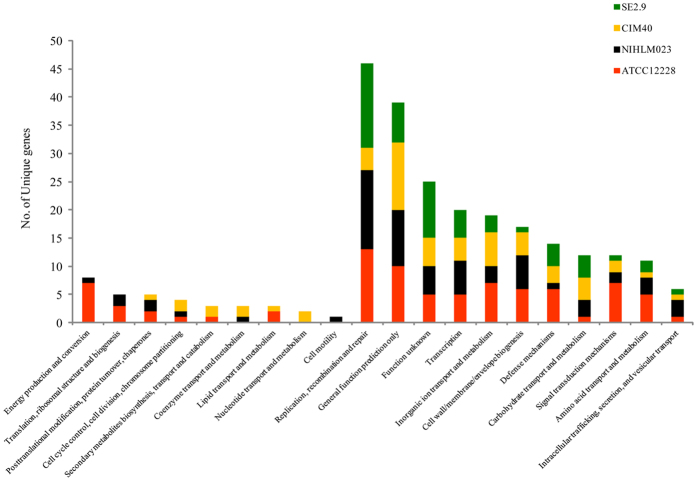
Distribution of COG based functional categories of unique genes of the *S. epidermidis* ATCC122278, NIHLM023, SE2.9 and CIM40 strains is illustrated in bar chart.

Such a large number of unique genes derived from pan-genome analysis, excited us to look at their functional classification so as to get the clue to their ecological diversification. Surprisingly, around 70% unique genes in each lineage were not assigned to any COG classes (Table S8). In four genomes viz., ATCC12228, NIHLM023, SE2.9 and CIM40, only 82, 64, 53 and 56 genes were classified into major and minor COG categories respectively (Fig. 4). The distribution of unique genes in COG functional categories is shown in Fig. 4.

Interestingly, out of 20 COG classes, animal (human/rodent) associated lineages have 17 COG classes while the plant associated RESE lineage have only 11 COG classes (Fig. 4, Table S8). Absence of almost half of the COG classes in RESE suggests major functional diversification in the form of unique genes. Even though RESE and rodent sub-lineages are phylogenetically linked (just 1% divergence at ANI level), there is drastic difference in number and types of unique genes COG classes between them. While RESE are less diverse with respect to unique genes COG classes, in contrast the rodent isolate has more number and type of COG classes similar to human isolates.

This was not surprising, and even expected as *S. epidermidis* isolates from rodent and human lineage have shared animal environment. But RESE are associated with plants which is altogether a contrasting habitat and distinct unique gene pool of RESE may be helping them to adapt as endophyte in plant niche. Interestingly, RESE lineage harbors relatively abundant unique pool of genes associated with replication, recombination, repair followed by unknown function. Reactive oxygen species (ROS) are produced as a product of plant metabolism and influence the expression of a number of genes and control different processes like growth, cell cycle, cell death, abiotic stress responses. Further, ROS are capable of causing cell damage by degradation of proteins, inactivation of enzymes and alterations in the gene^36^,^37^. The abundance of these genes associated in RESE strains might repair the damage caused by ROS produced and thereby protect their rice host plant to cope with diverse stresses and UV radiation.

### Understanding the genomic flux in the lineage that gave rise to RESE strains

One of the major sources of variable gene-pool or unique gene is horizontal gene transfer that is rampant in bacterial world^38^. In case of *S. epidermidis*, the percentage of unique genes with atypical GC content is more than 50% in all the four lineages. In major lineage A (ATCC12228) and RESE sub-lineage (SE2.9) it is 71% and 54% respectively (Table S4–S7). This further suggests that horizontal gene transfer is playing a major role in shaping the inter-strain diversity of *S. epidermidis* population of diverse phylogenetic and ecological origin. We also explored the location of such genomic flux, as horizontal gene transfer events of large chunk of DNA encoding genes are known to be important for adaptation or survival or pathogenicity.

**Figure 5:**
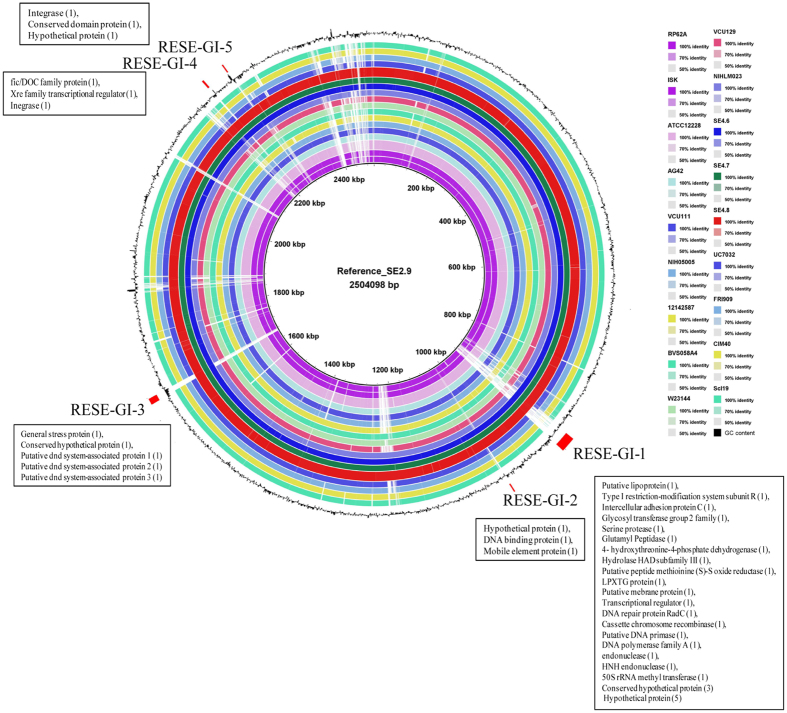
Circular representation of genomes of different lineages/sub-lineages representative strains of *S. epidermidis* on SE2.9 (RESE lineage) as reference. Colored rings represent different strains. The second to the outermost black circle represents the GC content (%) variations in the sequences. Location of regions unique to RESE lineage strains are marked by red in outer most circle. The ORFs encoded in these regions are indicated by their gene name as annotated in SE2.9 in boxes. Strains names with isolation source and accession numbers are listed in Table S2.

We carried out circular comparison of the genome of RESE isolates with a larger set of genomes representing human and rodent isolates (Fig. 5). The analysis revealed five genomic regions (RESE-GI-1 to RESE-GI-5) associated with the genome of RESE strains and might have played important role in the origin of RESE lineage (Fig. 5). We inspected sequence and function of each ORF in these regions to gain further insights. In RESE-GI-1, which is largest (24 kb) of the genomic regions, 8 ORFs encode hypothetical proteins. This further corroborated with our pan-genomic studies, where second largest class of unique genes were of unknown function (Fig. 4). Interestingly and importantly, in an earlier comparative study to identify genes unique to phytobacteria genomes, majority of them were found to encode hypothetical proteins^39^. Additionally, a group of ORFs with homology to hypothetical proteins in a genomic island have also been reported in *Enterobacter* sp. 638, which is well known plant growth promoting endophytic bacterium isolated from poplar^40^.

Despite rapid advances in the genetic studies to understand virulence function of phytopathogenic bacteria^41^,^42^, we know very less regarding genes required by a bacterium to establish as an endophyte. For example, one of the novel ways a plant associated bacteria protects plants is by producing anti-fungal metabolites like low molecular weight organic volatiles (VOCs)^22^,^23^. *S. epidermidis* isolated from potato, is reported to produce three VOCs that are antagonistic to phytopathogenic fungal pathogen^22^. Thus, there is a need for genetic screen, particularly the large number of hypothetical protein, to discern their possible role in production of VOCs and other such unique functions. Interestingly, one of the ORF in RESE-GI-1, that is unique to RESE genomes, encodes a methionine sulfoxide reductase (*msrA*). Plant parts like seeds are also known to have high levels of oxidation. This can lead to sulfation of methionine residues and ultimately damaging the protein and affecting its function^43^. A *msr* gene is also known to play role in protein repair and longevity of Arabidopsis seeds^43^. Incidentally, our RESE isolates are of seed origin and *msrA* gene might be helping the bacteria to overcome oxidation stress in plant seeds during maturation and dessication^44^,^45^.

Another ORF located after *msrA,* encode haloacid dehydrogenase (HAD). These HAD enzymes are implicated in protecting Ectocarpus from halogen-containing defence metabolites produced by kelp thali during its association as epiphyte or endophyte^46^,^47^,^48^. The third ORF present after *msrA* and HAD is homologous to 4-hydroxythreonine-4-phosphate dehydrogenase (*pdxA*), an intermediate enzyme in the synthesis of Vitamin B6 (Pyridoxine) (Fig. 5, Table S9). Vitamin B6 is known to protect bacteria from oxidative stress^49^. This gene has also been identified as gene unique to *Klebsiella pneumoniae* Kp342, an endophytic nitrogen-fixing diazotroph of *Zea mays*^50^. Presence of a cluster of three unique ORFs in RESE-GI-1 that encode functions required for tolerating stress and survival in plant is quite remarkable.

Plants along with its associated microbes are exposed to diverse environmental stresses including chemicals and radiations. To repair the DNA damage as a result of these stresses many genes get induced and their products are involved in DNA repair, replication and cell cycle control. RESE harbors several genes such as DNA primase, DNA polymerase family A, HNH endonuclease, DNA repair protein *radC* and general stress proteins that helps plants in overcoming diverse stresses by enhanced protection by DNA damage repair induced by UV radiation and general stress respectively. These fitness benefits conferred by RESE contribute to or are responsible for plant adaptation to stress. Studies indicates that members of the HNH family of endonucleases are homing endonucleases that transpose from one site into another and perform host cell repair mechanisms^51^,^52^,^53^,^54^. Moreover, the presence of *radC* in RESE is well corroborated with the earlier study done by Bertalan *et al.*^55^ where they found *radC* in all nine genomes of endophytic bacteria.

To develop as an endophyte, surface characteristics are one of the important factors for recognition and interaction of the bacterium with the plant host. Several genes related to surface components such as putative lipoprotein, glutamyl peptidase, serine protease, LPXTG protein, intercellular adhesion protein C and putative membrane protein are located in the RESE-GI-1 (Fig. 5, Table S9). Hydrolytic enzyme such as peptidase might help RESE to gain entry and for endophytic colonization^56^,^57^,^58^. Bacterial serine proteases are known for their diverse physiological functions associated with cell signaling, defense response and development^59^,^60^. Serine proteases are the largest category of proteases encoded in genome of *Pochonia chlamydosporia*, with more than half of the serine proteases were expressed during its endophytic lifestyle^58^. In one interesting study, Dunne and co-workers have showed improved biocontrol ability of mutant strains of *Stenotrophomonas maltophilia* with overproduced extracellular serine protease against *Pythium ultimum*^59^. Acquiring a serine protease by RESE strains might enable them to defend phytopathogens and thereby protecting rice plant health. Hence RESE-GI-1 is a major genomic island in RESE strains to enable them to gain entry, counter stress and survive and establish as a commensal in plants. At the same time the flux in RESE-GI-1 region (Fig. 5; Table S9) points towards their distinct selection pressure and evolutionary history of lineage that gave rice RESE strains.

Apart from RESE-GI-1, second largest genomic region of nearly 10 kb, RESE-GI-3, encode ORFs for functions involved in DNA phosphorothioation (known as the DND system). Interestingly, the DND system is one of the defense systems of prokaryotes^61^ and might protect RESE from phages that are unique to plant habitats. Additionally, one of the ORF in RESE-GI-3 codes for a general stress tolerance protein might help in endophytic lifestyle of these isolates. Like RESE-GI-2, other minor genomic islands harbor mobile elements, integrases along with gene(s) with function related to transcription regulation as in RESE-GI-4, DNA binding as in RESE-GI-2 and hypothetical protein as in RESE-GI-5 suggesting the on-going flux in RESE genomes and their adaptation to plant habitat (Fig. 5, Table S9) in a manner that is different from other human and animal associated *S. epidermidis* strains.

Plants represent one of the harshest habitats with array of biotic and abiotic stresses. Hence it was not surprising to find stress islands and distinct COG diversity of unique genes in RESE genomes that can benefit both host and inhabitant. It is also pertinent to note that from ecological point of view, there is scope for inter-strain recombination of genes required for stress tolerance or adaptation in novel habitat. This has significance with rising cases of nosocomial strains of *S. epidermidis* and its emergence as accidental human pathogen^2^. Even though, there is need for in depth functional studies of RESE genes using genetic and cellular approaches. At the same time we need to be cautious in exploiting such strains in agriculture.

## Conclusion

Whole genome based phylogenetic relationship and further comparative studies at population scale are much needed to understand origin and evolution of bacterial strains. In the present work, we have provided genome based evidence of diverse niches/lineages of *S. epidermidis*. Our genomic studies have revealed existence of a distinct sub-lineage of *S. epidermidis* isolated from surface sterilized rice seeds that are diverse from majority of human isolates but relatively closer with rodent isolates. At the same time further comparative genomics studies also revealed distinct genomic differences in RESE as a result of niche selective pressures. Our studies highlight an interesting example of infra-subspecies level genome diversification in *S. epidermidis* isolates from animal and plant origin.

## Materials and Methods

### Isolation and characterization of endophytic bacteria

Seeds were collected from rice fields located at Fazilka, Punjab (India) during harvesting month of November, 2012 and processed for seed surface sterilization in 3 independent batches using modified method of Liu *et al.*^62^. The hulls were removed from rice seeds using sterilized forceps, and the seeds (5 grams) were put in sterile falcon tubes and washed with 20 ml sterilized water for 1 min and then with 1% sodium hypochlorite solution for 5 min. The seeds were again washed with 75% ethanol for 1 min. After another wash with sterilized water five times, the surface sterilized rice seeds were crushed in sterile mortar and pestle and suspended in sterile saline solution (0.85% NaCl). The seeds suspension was incubated for 2 h at 28 °C under shaking condition. Then 100 μl of each of Direct, 10^−1^, 10^−2^, 10^−3^ and 10^−4^ dilution in sterile saline was plated in duplicates onto Nutrient agar (NA); King’s medium B (KMB); Glucose yeast chalk agar (GYCA); Tryptic soy agar (TSA); Peptone sucrose agar (PSA) supplemented with 0.01% cycloheximide. The confirmation of surface sterilization was conducted by spreading the last water wash as well as placing the washed seeds onto different media plates. The method for the isolation of endophytic bacteria from surface sterilized rice seeds is summarized in Fig. S1. Endophytic bacterial isolates were identified and confirmed on the basis of 16S rRNA gene sequencing and analysis, performed using web based tool EzTaxon-e (http://www.ezbiocloud.net/eztaxon)^63^ prior to whole genome sequencing. Complete 16S rRNA genes of all four *S. epidermidis* SE2.9, SE4.6, SE4.7 and SE4.8 is submitted to NCBI with GenBank accession no. KM877504, KM877505, KM877506 and KM877507 respectively.

### Whole genome sequencing (WGS) and data collection

“Type strain” of *S. epidermidis* MTCC3382(T) [=NCTC11047(T) = JCM2414(T) = ATCC14990(T)] was obtained from Microbial Type Culture Collection and Gene Bank (MTCC), Chandigarh, India. The strain was confirmed on the basis of 16S rRNA gene sequence analysis. Strains were cultured in Nutrient Broth (NB) medium with shaking at 150 rpm and 28 °C for 18 hours. Genomic DNA was isolated using Zymo ZR- Fungal/bacterial DNA isolation kit (Zymo Research Corporation, Orange, CA). The quality and concentration of the isolated genomic DNA was assessed using agarose gel electrophoresis, NanoDrop spectrophotometer ND-1000 (Thermo Fisher Scientific, USA) and Qubit 2.0 fluorometer (Invitrogen, USA). The input of 1 μg of genomic DNA from each sample was taken and the standard protocol for the Nextera XT DNA sample preparation kit was used for library construction. The purified fragmented DNA was used as a template for a limited cycle PCR using Nextera primers and index adaptors. Cluster generation and sequencing of libraries were performed on the Illumina MiSeq platform (Illumina, San Diego, CA) with a 2 × 250 paired-end run.

### Genome sequence analysis, assembly and annotation

Demultiplexing, fastq generation and adapter trimming steps in sequence reads were automatically performed by Illumina- MiSeq software. The paired-end raw reads containing fastq files were assembled into contigs using CLC Genomics Workbench software version 7.0.3 (CLC Inc, Aarhus, Denmark) and total number of contigs, genome size, G+C content and total coverage was obtained. The draft genomes were annotated using Prokaryotic Genomes Automatic Annotation Pipeline (PGAAP) at NCBI (http://www.ncbi.nlm.nih.gov/genomes/static/Pipeline.html) and Rapid Annotation System Technology (RAST) pipeline^64^.

### Phylogenomic analysis

To establish the relatedness among the *S. epidermidis* genomes sequences, ANI was performed with phylogenetic representatives of *S. epidermidis* and MTCC3382(T) genome using JSpecies v1.2.1^32^ and visualized as heatmap using software Gene-e (http://www.broadinstitute.org/cancer/software/GENE-E/). Further, dDDH of “type strain” MTCC3382(T) against RESE and representatives *S. epidermidis* strains was calculated using GGDC 2.0 server (http://ggdc.dsmz.de/distcalc.php) by means of genome-to-genome sequence comparison^33^.

To determine the relatedness of *S. epidermidis* isolates to one another, a whole-genome phylogeny was performed using 31 universal housekeeping genes^65^. These housekeeping genes from RESE and other *S. epidermidis* genomes available in NCBI Genbank were retrieved, concatenated and aligned. A maximum likelihood tree was constructed on 31 housekeeping genes using General Time reversible (GTR) model, Gamma distributed with Invariant sites (G + I) method with 500 bootstrap replications MEGA v-6^66^.

### Comparative genomics

Pan-genomes were built to estimate the number of shared genes (core genome) and unique genes (accessory or variable genome), using Pan-Genome Analysis Pipeline^67^. This multiparanoid based algorithm searches for homologs/orthologs in multiple genomes considering local matched region to be not less than 25% of the longer gene protein sequence and global matched region not less than 50% of the longer gene protein sequence. A minimum score value of 50 and an E-value of less than 1 × 10^−8^ respectively, were used as cutoffs. Further unique genes were functionally characterized using RAST and classified to a specific Clusters of Orthologous Groups (COG) family by searching against the COGs database using online tool WebMGA^68^.

Genome level differences between RESE genomes with other groups were analysed using BRIG-0.95^69^. Circular genome maps was generated using reference and query genome sequences in a set of concentric rings colored according to BLAST identity. BRIG generated regions of interest were re-annotated using RAST pipeline^64^ and re-inspected for homology by BlastP and assessment of function.

### Nucleotide sequence accession numbers

The Whole Genome Shotgun project of *S. epidermidis* MTCC3382(T), SE2.9, SE4.6, SE4.7 and SE4.8 strains have been deposited at DDBJ/EMBL/GenBank under the accession number LILE00000000, JRVN00000000, JRVO00000000, JRVP00000000 and JRVQ00000000 respectively.

## Additional Information

**How to cite this article**: Chaudhry, V. and Patil, P. B. Genomic investigation reveals evolution and lifestyle adaptation of endophytic *Staphylococcus epidermidis*. *Sci. Rep.* **6**, 19263; doi: 10.1038/srep19263 (2016).

## Supplementary Material

Supplementary Information

## Figures and Tables

**Table 1:** General Genomic features of *S. epidermidis* “type strain” MTCC3382(T) and RESE genomes sequenced in this study.

	Value for indicated strains				
Parameter	MTCC3382 (T)	SE2.9	SE4.6	SE4.7	SE4.8
Genome size (bp)	2,383,824	2,504,098	2,481,553	2,489,127	2,484,083
GC content (%)	32.1	31.9	31.9	31.8	31.9
No. of genes	2,253	2,393	2,367	2,379	2,376
No. of rRNA	7	8	5	7	7
No. of tRNA	56	55	56	57	56
NCBI Accession no.	LILE00000000	JRVN00000000	JRVO00000000	JRVP00000000	JRVQ00000000

**Table 2:** Average Nucleotide Identity (ANI) and Digital DNA-DNA hybridization (dDDH) in percent between *S. epidermidis* “type strain” MTCC3382(T) and RESE genomes.

S.No.	Strains	*S. epidermidis* MTCC3382 (T)	
		ANI	dDDH
1.	*S. epidermidis* SE2.9	97.02	74.9
2.	*S. epidermidis* SE4.6	97.04	75
3.	*S. epidermidis* SE4.7	97.03	74.9
4.	*S. epidermidis* SE4.8	97.03	74.9
