# Supplementary material for: Genomic investigation reveals evolution and lifestyle adaptation of endophytic Staphylococcus epidermidis
Source: Sci Rep. 2016 Jan 13;6:19263. doi: 10.1038/srep19263 (PMC4713051; doi:10.1038/srep19263)
Supplement: Supplementary Information [file srep19263-s1.pdf]

Genomic investigation reveals evolution and lifestyle adaptation of endophytic *Staphylococcus epidermidis*

Vasvi Chaudhry and Prabhu B. Patil*

Supplementary information

Figure S1: Stepwise procedure for the isolation of endophytic bacteria from surface sterilized rice seeds is depicted in figure.

Table S1: De novo assembly statistics of *S. epidermidis* "type strain" MTCC3382(T) and RESE sequenced in this study.

Table S2: List of all *S. epidermidis* strains with isolation source and accession numbers used for analysis in the present study.

Table S3: Digital DNA-DNA hybridization (dDDH) in percent between RESE (SE2.9 as reference) and different lineages/sub-lineages *S. epidermidis* (query genomes).

Table S4: List of unique genes, their putative function and GC Content (%) of *S. epidermidis* ATCC12228.

Table S5: List of unique genes, their putative function and GC Content (%) of *S. epidermidis* NIHLM023.

Table S6: List of unique genes, their putative function and GC Content (%) of *S. epidermidis* SE2.9.

Table S7: List of unique genes, their putative function and GC Content (%) of *S. epidermidis* CIM40.

Table S8: COG based functional categories of unique genes in *S. epidermidis* ATCC12228, NIHLM023, SE2.9 and CIM40.

Table S9: List of unique genes in RESE annotated in hyper-variable regions generated by BRIG, their putative function and GC Content (%).

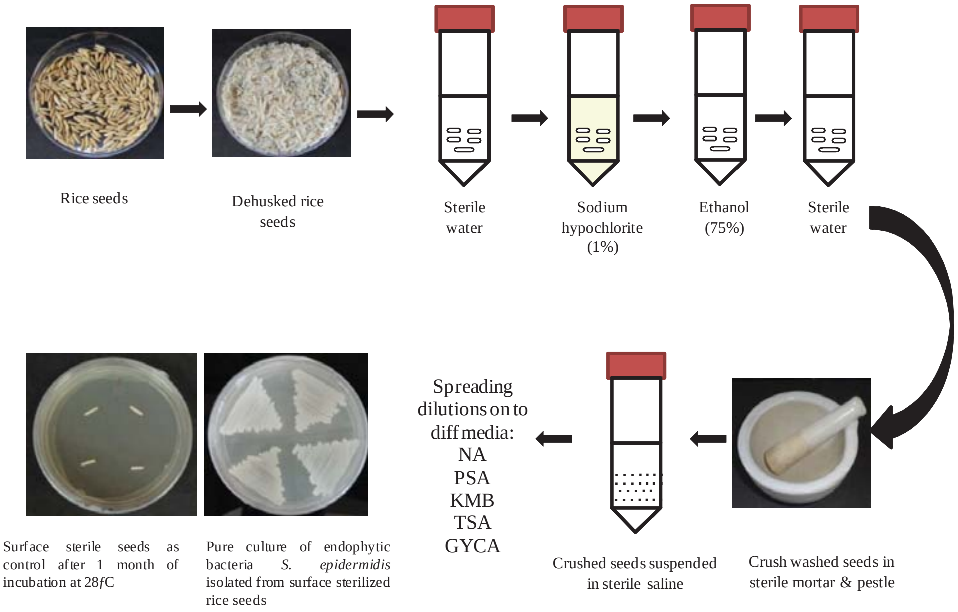

Figure S1: Stepwise procedure for the isolation of endophytic bacteria from surface sterilized rice seeds is depicted in figure.

Table S1: De novo assembly statistics of *S. epidermidis* "type strain" MTCC3382(T) and RESE sequenced in this study.

S. No	Strains	Total no. of reads	Average read length (bp)	Total data generated (bp)	Total Contigs	N50	Average contig size (bp)	Genome size (bp)	Coverage
1.	***Staphylococcus epidermidis* MTCC3382(T)**	3,114,396	130.73	407,131,973	85	70,355	28,044	2,383,824	170X
2.	***Staphylococcus epidermidis* SE2.9**	1,852,926	232.93	431,609,649	38	175,049	65,897	2,504,098	172X
3.	***Staphylococcus epidermidis* SE4.6**	967,084	162.4	157,052,145	85	65,693	29,194	2,481,553	63X
4.	***Staphylococcus epidermidis* SE4.7**	1,271,238	159.37	202,598,711	77	95,985	32,326	2,489,127	81X
5.	***Staphylococcus epidermidis* SE4.8**	889,566	159.72	142,083,153	124	48,988	20,032	2,484,083	57X

Table S2: List of all *S. epidermidis* strains used for analysis in the present study

	Strain	Habitat/Host	Site	Geographical location/country	Isolation year	Phenotype	Genbank Accession no.
1	*Staphylococcus epidermidis* M23864:W2(grey)	Human	Skin				NZ_ADMU00000000
2	*Staphylococcus epidermidis* M0881	Human	Blood	USA	2004		NZ_AOAJ00000000
3	*Staphylococcus epidermidis* NIH04003	Human	Blood	USA			NZ_AKHJ00000000
4	*Staphylococcus epidermidis* NIH06004	Human	Blood	USA			NZ_AKHH00000000
5	*Staphylococcus epidermidis* NIH08001	Human	Blood	USA			NZ_AKHG00000000
6	*Staphylococcus epidermidis* VCU045	Human airways		USA			NZ_AFEI00000000
7	*Staphylococcus epidermidis* VCU037	Human airways		USA			NZ_AFTY00000000
8	*Staphylococcus epidermidis* M0026	Human	Blood	USA	2003		NZ_JBVX00000000
9	*Staphylococcus epidermidis* RP62A	Human		USA		Pathogen, Biofilm forming	NC_002976
10	*Staphylococcus epidermidis* NIHLM088	Human	nare	USA			NZ_AKGJ00000000
11	*Staphylococcus epidermidis* 41tr	Human		Russia	2011		NZ_APHU00000000
12	*Staphylococcus epidermidis* 528m	Human		Russia	2011		NZ_APHT00000000
13	*Staphylococcus epidermidis* NIH051668	Human	Lung biopsy	USA			NZ_AKHK00000000
14	*Staphylococcus epidermidis* NIH04008	Human	Blood	USA			NZ_AKHF00000000
15	*Staphylococcus epidermidis* VCU041	Human airways		USA			NZ_AHKX00000000
16	*Staphylococcus epidermidis* NIHLM008	Human	alar crease	USA			NZ_AKHA00000000
17	*Staphylococcus epidermidis* VCU127	Human airways		USA			NZ_AHLH00000000
18	*Staphylococcus epidermidis* NIHLM003	Human	umbilicus	USA			NZ_AKHB00000000
19	*Staphylococcus epidermidis* NIHLM070	Human	alar crease	USA			NZ_AKGL00000000
20	*Staphylococcus epidermidis* E13A	Human					NZ_AURC00000000
21	*Staphylococcus epidermidis* MTCC3382(T) =NCTC11047 =ATCC 14990	Human	Nose				LILE00000000
22	*Staphylococcus epidermidis* IS-250	Human		USA		Pathogen	NZ_AJJR00000000
23	*Staphylococcus epidermidis* IS-K	Human		USA		Pathogen	NZ_AJJS00000000
24	*Staphylococcus epidermidis* VCU028	Human airways		USA			NZ_AFEH00000000
25	*Staphylococcus epidermidis* VCU081	Human airways		USA			NZ_AHLU00000000
26	*Staphylococcus epidermidis* Scl25	Wild mouse	skin	Germany	2012		NZ_ATDC00000000
27	*Staphylococcus epidermidis* Scl31	Wild mouse	skin	Germany	2012		NZ_ATDD00000000
28	*Staphylococcus epidermidis* VCU125	Human airways		USA			NZ_AHLF00000000
29	*Staphylococcus epidermidis* AU12-03	Human	intravascular catheter	Australia			NZ_AMCS00000000
30	*Staphylococcus epidermidis* SK135	Human	skin	USA			NZ_ADEY00000000
31	*Staphylococcus epidermidis* BCM-HMP0060	Human	skin	USA			NZ_ACHE00000000
32	*Staphylococcus epidermidis* VCU109	Human airways		USA			NZ_AFUA00000000
33	*Staphylococcus epidermidis* NIH05003	Human	Blood	USA			NZ_AKHI00000000
34	*Staphylococcus epidermidis* NIHLM020	Human	axilla	USA			NZ_AKGW00000000
35	*Staphylococcus epidermidis* NIHLM018	Human	umbilicus	USA			NZ_AKGY00000000
36	*Staphylococcus epidermidis* VCU065	Human airways		USA			NZ_AHKZ00000000
37	*Staphylococcus epidermidis* strain 1457	Human	Central	Germany	1990		NZ_JMID00000000

			veneous cathetar				
38	*Staphylococcus epidermidis* ATCC 12228	Human	Skin			Mildly infectious, Non-Biofilm forming	NC_004461
39	*Staphylococcus epidermidis* VCU057	Human airways		USA			NZ_AHKY00000000
40	*Staphylococcus epidermidis* strain 8400	Human	blood	Germany	1990		NZ_JMIF00000000
41	*Staphylococcus epidermidis* VCU036	Human airways		USA			NZ_JHUA00000000
42	*Staphylococcus epidermidis* VCU105	Human airways		USA			NZ_AFTZ00000000
43	*Staphylococcus epidermidis* AG42	Sheep rumen					NZ_JNLI00000000
44	*Staphylococcus epidermidis* NIHLM039	Human	retroauricu lar crease	USA			NZ_AKGS00000000
45	*Staphylococcus epidermidis* VCU123	Human airways		USA			NZ_AHLE00000000
46	*Staphylococcus epidermidis* NIHLM001	Human	alar crease	USA			NZ_AKHC00000000
47	*Staphylococcus epidermidis* VCU144	Human airways		USA			NZ_AFED00000000
48	*Staphylococcus epidermidis* NIHLM021	Human	axilla	USA			NZ_AKGV00000000
49	*Staphylococcus epidermidis* VCU111	Human		USA			NZ_JHUB00000000
50	*Staphylococcus epidermidis* VCU117	Human airways		USA			NZ_AHLA00000000
51	*Staphylococcus epidermidis* VCU126	Human airways		USA			NZ_AHLG00000000
52	*Staphylococcus epidermidis* NIHLM067	Human	axilla	USA			NZ_AKGM00000000
53	*Staphylococcus epidermidis* NIHLM049	Human	gluteal crease	USA			NZ_AKGQ00000000
54	*Staphylococcus epidermidis* VCU050	Human airways					NZ_JHQC00000000
55	*Staphylococcus epidermidis* NIH05001	Human	Blood	USA			NZ_AKHE00000000
56	*Staphylococcus epidermidis* strain 1057	Human	central veneous catheter	Germany	1990		NZ_JMIE00000000
57	*Staphylococcus epidermidis* NIH05005	Human	Blood	USA			NZ_AKHD00000000
58	*Staphylococcus epidermidis* VCU013	Human		USA			NZ_JHTZ00000000
59	*Staphylococcus epidermidis* VCU014	Human airways		USA			NZ_JHQB00000000
60	*Staphylococcus epidermidis* VCU120	Human airways		USA			NZ_AHLC00000000
61	*Staphylococcus epidermidis* NIH051475	Human	Joint fluid	USA			NZ_AKHL00000000
62	*Staphylococcus epidermidis* 12142587	Human					NZ_AMSJ00000000
63	*Staphylococcus epidermidis* NIHLM040	Human	retroauricu lar crease	USA			NZ_AKGR00000000
64	*Staphylococcus epidermidis* VCU071	Human airways		USA			NZ_AGUB00000000
65	*Staphylococcus epidermidis* BVS058A4	Human	Urogenital tract , Vagina	USA			NZ_AGZV00000000
66	*Staphylococcus epidermidis* NIHLM087	Human	nare	USA			NZ_AKGK00000000
67	*Staphylococcus epidermidis* NIHLM095	Human	gluteal crease	USA			NZ_AKGI00000000
68	*Staphylococcus epidermidis* NIHLM061	Human	nare	USA			NZ_AKGN00000000
69	*Staphylococcus epidermidis* W23144	Human	skin	USA			NZ_ACJC00000000
70	*Staphylococcus epidermidis* NIHLM015	Human	manubriu m	USA			NZ_AKGZ00000000
71	*Staphylococcus epidermidis* VCU129	Human airways		USA			NZ_AHLJ00000000

72	*Staphylococcus epidermidis* NIHLM037	Human	glabella	USA			NZ_AKGT00000000
73	*Staphylococcus epidermidis* VCU118	Human airways		USA			NZ_AHLB00000000
74	*Staphylococcus epidermidis* NIHLM023	Human	toe web	USA			NZ_AKGU00000000
75	*Staphylococcus epidermidis* VCU128	Human airways		USA			NZ_AHLI00000000
76	*Staphylococcus epidermidis* NIHLM053	Human	hypothenar palm	USA			NZ_AKGP00000000
77	*Staphylococcus epidermidis* NIHLM057	Human	occiput	USA			NZ_AKGO00000000
78	*Staphylococcus epidermidis* SE4.7	Rice	Seed interior	India			JRVP00000000
79	*Staphylococcus epidermidis* SE4.8	Rice	Seed interior	India			JRVQ00000000
80	*Staphylococcus epidermidis* SE4.6	Rice	Seed interior	India			JRVO00000000
81	*Staphylococcus epidermidis* SE2.9	Rice	Seed interior	India			JRVN00000000
82	*Staphylococcus epidermidis* UC7032	Pig	cured meat				NZ_ARWU00000000
83	*Staphylococcus epidermidis* NIHLM031	Human	plantar heel	USA			NZ_AKGX00000000
84	*Staphylococcus epidermidis* FRI909	Human		USA			NZ_AENR00000000
85	*Staphylococcus epidermidis* APO27	Wild mouse	skin	Germany	2012		NZ_ATCU00000000
86	*Staphylococcus epidermidis* APO35	Wild mouse	skin				NZ_ATCV00000000
87	*Staphylococcus epidermidis* CIM28	Wild mouse	skin				NZ_ATDF00000000
88	*Staphylococcus epidermidis* CIM40	Wild mouse	skin		2012		NZ_ATCW00000000
89	*Staphylococcus epidermidis* MC16	Wild mouse	skin				NZ_ATCX00000000
90	*Staphylococcus epidermidis* MC28	Wild mouse	skin		2012		NZ_ATCZ00000000
91	*Staphylococcus epidermidis* MC19	Wild mouse	skin		2012		NZ_ATCY00000000
92	*Staphylococcus epidermidis* Scl19	Wild mouse	skin		2012		NZ_ATDA00000000

Table S3: Digital DNA-DNA hybridization (dDDH) in percent between RESE (SE2.9 as reference) and different lineages/sub-lineages *S. epidermidis* (query genomes).

Reference genome	Query genome	dDDH
SE2.9	SE BVSO58A4	73
SE2.9	SE 12142587	73.4
SE2.9	SE NIH5005	74.4
SE2.9	SE AG42	74.6
SE2.9	SE MTCC3382(T)	74.9
SE2.9	SE VCU111	74.8
SE2.9	SE ISK	74.9
SE2.9	SE ATCC12228	74.9
SE2.9	SE RP62A	76.2
SE2.9	SE VCU129	89.8
SE2.9	SE W23144	90
SE2.9	SE CIM40	90.4
SE2.9	SE Scl19	90.4
SE2.9	SE NIHLM023	90.6
SE2.9	SE UC7032	90.9
SE2.9	SE FRI909	91.6
SE2.9	SE2.9	100
SE2.9	SE4.6	100
SE2.9	SE4.7	100
SE2.9	SE4.8	100

Table S4: List of unique genes, their putative function and GC Content (%) of *S. epidermidis* ATCC12228.

Cluster Id	Gene Id	Function	GC content
1	27466935	hypothetical protein	23.66%
2	27466942	hypothetical protein	19.67%
3	27466943	CDP-glycerol:poly(glycerophosphate) glycerophosphotransferase (EC 2.7.8.12)	25.30%
4	27466944	Spermidine N1-acetyltransferase (EC 2.3.1.57)	27.11%
5	27466945	hypothetical protein	22.48%
6	27466946	Putative membrane protein	29.93%
7	27466950	hypothetical protein	33.33%
8	27466951	FIG01108299: hypothetical protein	35.90%
9	27466955	Cassette chromosome recombinase A	34.59%
10	27466957	FIG01108228: hypothetical protein	34.68%
11	27466958	FIG01108557: hypothetical protein	25.02%
12	27466959	Abi-alpha protein	28.57%
13	27466960	abortive phage resistance protein	27.47%
14	27466961	Type I restriction-modification system, restriction subunit R (EC 3.1.21.3)	25.00%
15	27466962	Type I restriction-modification system, specificity subunit S (EC 3.1.21.3)	28.26%
16	27466963	Transposase for IS1272	25.00%
17	27466964	Spermidine N1-acetyltransferase (EC 2.3.1.57)	27.11%
18	27466965	hypothetical protein	22.48%
19	27466966	Putative membrane protein	29.68%
20	27466967	hypothetical protein	31.48%
21	27466972	hypothetical protein	31.18%
22	27466973	FIG01108299: hypothetical protein	36.75%
23	27466975	Cassette chromosome recombinase A	33.19%
24	27466976	FIG01108399: hypothetical protein	32.82%
25	27466977	Cassette chromosome recombinase A	30.83%
26	27466978	Zinc transporter, ZIP family	35.56%
27	27466979	hypothetical protein	39.39%
28	27466980	Lead, cadmium, zinc and mercury transporting ATPase (EC 3.6.3.3) (EC 3.6.3.5); Copper-translocating P-type ATPase (EC 3.6.3.4)	34.94%
29	27466982	transposase	27.68%
30	27466983	transposase	27.64%
31	27466984	transposase	22.22%
32	27466986	hypothetical protein	24.56%
33	27466987	hypothetical protein	22.97%
34	27466988	hypothetical protein	32.91%
35	27466990	Lipoprotein signal peptidase (EC 3.4.23.36)	35.19%
36	27466992	Zinc transporter, ZIP family	37.11%
37	27466993	Cadmium-transporting ATPase (EC 3.6.3.3)	37.61%
38	27466994	Mobile element protein	29.18%
39	27466995	Dihydrolipoamide dehydrogenase (EC 1.8.1.4)	39.93%
40	27466996	Cadmium resistance protein	34.39%
41	27466998	similar to glutathione reductase	41.04%
42	27467000	FIG01109440: hypothetical protein	38.42%
43	27467001	FIG01108609: hypothetical protein	42.29%
44	27467002	mercuric transport protein	44.83%
45	27467003	Mercuric ion reductase (EC 1.16.1.1)	46.23%
46	27467004	Organomercurial lyase (EC 4.99.1.2)	53.15%
47	27467005	FIG01109191: hypothetical protein	39.53%

48	27467006	hypothetical protein	42.59%
49	27467007	hypothetical protein	37.94%
50	27467009	type I restriction enzyme R protein	29.73%
51	27467010	hypothetical protein	28.74%
52	27467012	ABC transporter, ATP-binding protein	28.67%
53	27467013	ABC-type multidrug transport system permease component	30.23%
54	27467014	Bifunctional protein: zinc-containing alcohol dehydrogenase; quinone oxidoreductase (NADPH:quinone reductase) (EC 1.1.1.-); Similar to arginate lyase	31.33%
55	27467015	hypothetical protein	28.22%
56	27467016	hypothetical protein	24.92%
57	27467017	hypothetical protein	25.51%
58	27467018	Mobile element protein	28.03%
59	27467020	Carbamate kinase (EC 2.7.2.2)	35.59%
60	27467021	Ornithine carbamoyltransferase (EC 2.1.3.3)	33.73%
61	27467022	Transcriptional regulator ArcR essential for anaerobic expression of the ADI pathway, Crp/Fnr family	23.77%
62	27467023	Arginine/ornithine antiporter ArcD	33.33%
63	27467024	Arginine deiminase (EC 3.5.3.6)	33.25%
64	27467025	Arginine pathway regulatory protein ArgR, repressor of arg regulon	25.28%
65	27467026	type I restriction-modification system endonuclease	32.14%
66	27467027	hypothetical protein	21.88%
67	27467030	FIG01110863: hypothetical protein	38.32%
68	27467031	hypothetical protein	31.07%
69	27467033	radical SAM domain protein	27.53%
70	27467034	hypothetical protein	22.46%
71	27467035	ABC transporter ATP-binding protein	28.59%
72	27467036	FIG01110247: hypothetical protein	25.13%
73	27467037	FIG01109362: hypothetical protein	23.37%
74	27467038	response regulator	27.35%
75	27467039	two-component sensor histidine kinase	26.92%
76	27467040	hypothetical protein	25.53%
77	27467043	immunodominant antigen B	30.19%
78	27467049	Disulfide bond regulator	30.08%
79	27467062	FIG01108158: hypothetical protein	26.85%
80	27467068	hypothetical protein	25.23%
81	27467071	hypothetical protein	25.00%
82	27467073	hypothetical protein	30.30%
83	27467076	hypothetical protein	23.53%
84	27467077	hypothetical protein	23.81%
85	27467078	3-oxoacyl-[acyl-carrier protein] reductase (EC 1.1.1.100)	35.29%
86	27467079	Mobile element protein	32.80%
87	27467082	Glycerol-3-phosphate transporter	36.16%
88	27467083	DNA-binding response regulator, AraC family	32.54%
89	27467084	Two-component sensor histidine kinase	34.70%
90	27467085	Ferric iron ABC transporter, iron-binding protein	36.21%
91	27467086	Minor teichoic acid biosynthesis protein GgaB	24.76%
92	27467087	hypothetical protein	20.45%
93	27467088	Minor teichoic acid biosynthesis protein GgaB	25.59%
94	27467089	hypothetical protein	22.50%
95	27467091	FIG01109073: hypothetical protein	34.80%
96	27467092	hypothetical protein	39.22%
97	27467093	Virulence-associated cell-wall-anchored protein SasG (LPXTG motif), binding to squamous nasal epithelial cells	39.71%
98	27467094	hypothetical protein	39.66%

99	27467096	hypothetical protein	24.73%
100	27467104	FIG01108606: hypothetical protein	28.57%
101	27467108	hypothetical protein	34.95%
102	27467110	hypothetical protein	37.61%
103	27467117	Glutamate synthase [NADPH] large chain (EC 1.4.1.13)	30.20%
104	27467118	hypothetical protein	21.74%
105	27467122	hypothetical protein	34.41%
106	27467125	hypothetical protein	33.33%
107	27467151	hypothetical protein	30.11%
108	27467164	hypothetical protein	18.75%
109	27467175	Mobile element protein	33.33%
110	27467183	hypothetical protein	20.95%
111	27467197	hypothetical protein	29.52%
112	27467198	hypothetical protein	30.63%
113	27467220	hypothetical protein	35.00%
114	27467246	hypothetical protein	27.27%
115	27467251	hypothetical protein	35.29%
116	27467252	Arsenic efflux pump protein	29.07%
117	27467254	hypothetical protein	30.30%
118	27467263	hypothetical protein	25.40%
119	27467273	Mobile element protein	32.61%
120	27467295	FIG01109785: hypothetical protein	24.11%
121	27467296	hypothetical protein	23.42%
122	27467311	hypothetical protein	22.86%
123	27467312	hypothetical protein	27.21%
124	27467334	hypothetical protein	26.13%
125	27467376	hypothetical protein	32.35%
126	27467387	hypothetical protein	27.27%
127	27467394	hypothetical protein	31.18%
128	27467398	hypothetical protein	32.79%
129	27467400	hypothetical protein	32.32%
130	27467401	Mobile element protein	31.62%
131	27467413	Mobile element protein	29.06%
132	27467416	hypothetical protein	19.19%
133	27467458	hypothetical protein	27.96%
134	27467486	Degenerate integrase, superantigen-encoding pathogenicity islands SaPI	35.74%
135	27467488	FIG01108889: hypothetical protein	28.21%
136	27467490	hypothetical protein	19.79%
137	27467493	hypothetical protein	27.18%
138	27467507	hypothetical protein	17.83%
139	27467508	Mobile element protein	32.48%
140	27467515	hypothetical protein	34.41%
141	27467529	hypothetical protein	26.04%
142	27467530	hypothetical protein	32.22%
143	27467547	Mobile element protein	29.46%
144	27467570	Mobile element protein	29.47%
145	27467579	hypothetical protein	35.35%
146	27467586	Mobile element protein	32.48%
147	27467589	PaaD-like protein (DUF59) involved in Fe-S cluster assembly	34.10%
148	27467641	Serine protease, DegP/HtrA, do-like (EC 3.4.21.-)	33.46%
149	27467648	hypothetical protein	19.79%
150	27467652	putative fatty acid desaturase	27.19%

151	27467657	hypothetical protein	17.20%
152	27467660	hypothetical protein	28.13%
153	27467738	hypothetical protein	28.33%
154	27467747	hypothetical protein	20.10%
155	27467792	hypothetical protein	28.70%
156	27467837	FIG01108412: hypothetical protein	28.47%
157	27467838	hypothetical protein	21.93%
158	27467864	ribosome-binding factor A	30.79%
159	27467879	Molybdopterin binding motif, CinA N-terminal domain / C-terminal domain of CinA type S	30.60%
160	27467907	Portal protein, truncation	27.92%
161	27467909	FIG01107943: hypothetical protein	21.88%
162	27467911	FIG01109296: hypothetical protein	28.04%
163	27467919	sensor histidine kinase	26.59%
164	27467939	hypothetical protein	27.91%
165	27467940	SOS-response repressor and protease LexA (EC 3.4.21.88)	30.77%
166	27467962	Mobile element protein	32.48%
167	27468000	ABC transporter protein	24.97%
168	27468001	Protein msa (Modulator of sarA)	22.63%
169	27468002	hypothetical protein	21.21%
170	27468016	hypothetical protein	27.03%
171	27468019	hypothetical protein	22.52%
172	27468030	hypothetical protein	22.22%
173	27468047	Putative Staphylococcal surface anchored protein	34.66%
174	27468067	hypothetical protein	27.27%
175	27468079	Glycerol-3-phosphate dehydrogenase [NAD(P)+] (EC 1.1.1.94)	39.51%
176	27468082	SSU ribosomal protein S1p	33.13%
177	27468091	hypothetical protein	28.57%
178	27468107	hypothetical protein	25.00%
179	27468137	truncated transposase	27.04%
180	27468141	Shikimate kinase I (EC 2.7.1.71)	30.30%
181	27468157	hypothetical protein	18.63%
182	27468234	hypothetical protein	22.55%
183	27468237	Mobile element protein	31.62%
184	27468280	hypothetical protein	20.20%
185	27468304	transposase	33.33%
186	27468317	hypothetical protein	21.33%
187	27468338	hypothetical protein	21.21%
188	27468363	hypothetical protein	25.83%
189	27468372	Mobile element protein	35.90%
190	27468385	Integrase, phage associated	29.13%
191	27468387	hypothetical protein	29.52%
192	27468388	Phage lysin, N-acetylmuramoyl-L-alanine amidase (EC 3.5.1.28)	37.00%
193	27468389	hypothetical protein	27.96%
194	27468390	Phage integrase	28.69%
195	27468391	hypothetical protein	33.33%
196	27468401	ATPase involved in DNA repair	22.45%
197	27468404	hypothetical protein	35.83%
198	27468440	hypothetical protein	36.19%
199	27468454	Hypothetical protein SAV1854	28.57%
200	27468457	Mobile element protein	35.04%
201	27468462	hypothetical protein	47.37%
202	27468474	hypothetical protein	36.88%

203	27468523	FIG01107859: hypothetical protein	29.71%
204	27468527	FIG01109633: hypothetical protein	27.14%
205	27468528	FIG01109064: hypothetical protein	24.55%
206	27468529	Tn554-related, transposase C	24.32%
207	27468530	Tn554-related, transposase B	27.12%
208	27468531	Tn554-related, transposase A	25.87%
209	27468538	Thioredoxin	31.37%
210	27468552	delta-hemolysin	26.92%
211	27468559	hypothetical protein	26.88%
212	27468560	Sucrose operon repressor ScrR, LacI family	27.08%
213	27468581	hypothetical protein	40.54%
214	27468582	hypothetical protein	47.37%
215	27468583	hypothetical protein	23.02%
216	27468601	hypothetical protein	31.88%
217	27468673	hypothetical protein	47.37%
218	27468691	alkaline shock protein 23	31.42%
219	27468731	LSU ribosomal protein L14p (L23e)	38.06%
220	27468757	transposase	30.48%
221	27468771	Formate dehydrogenase chain D (EC 1.2.1.2)	33.08%
222	27468772	Formate dehydrogenase chain D (EC 1.2.1.2)	28.92%
223	27468777	hypothetical protein	23.23%
224	27468817	Mobile element protein	33.33%
225	27468827	hypothetical protein	25.00%
226	27468828	hypothetical protein	20.97%
227	27468834	hypothetical protein	29.41%
228	27468836	hypothetical protein	27.96%
229	27468840	hypothetical protein	33.33%
230	27468849	hypothetical protein	24.36%
231	27468871	FIG01109096: hypothetical protein	37.88%
232	27468872	FIG01109314: hypothetical protein	31.11%
233	27468873	Copper resistance protein D	33.99%
234	27468874	Copper resistance protein D	29.87%
235	27468875	Copper resistance protein D	34.32%
236	27468898	hypothetical protein	24.07%
237	27468900	Mobile element protein	33.33%
238	27468908	Mobile element protein	31.29%
239	27468912	hypothetical protein	32.56%
240	27468924	hypothetical protein	37.04%
241	27468944	hypothetical protein	32.78%
242	27468955	FIG01107932: hypothetical protein	31.97%
243	27468957	DNA/RNA helicase of DEAD/DEAH box family	26.52%
244	27468958	Mutator mutT protein (7,8-dihydro-8-oxoguanine-triphosphatase) (EC 3.6.1.-)	30.88%
245	27468967	hypothetical protein	32.32%
246	27468996	transposase	33.33%
247	27469002	hypothetical protein	20.95%
248	27469018	hypothetical protein	33.33%
249	27469019	hypothetical protein	41.99%
250	27469041	hypothetical protein	26.95%
251	27469045	hypothetical protein	26.67%
252	27469052	Mobile element protein	32.48%
253	27469055	hypothetical protein	27.78%
254	27469056	FIG01109184: hypothetical protein	33.64%

255	27469067	hypothetical protein	24.64%
256	27469071	Mobile element protein	32.67%
257	27469073	hypothetical protein	23.96%
258	27469082	Catalyzes the cleavage of p-aminobenzoyl-glutamate to p-aminobenzoate and glutamate, subunit A	38.10%
259	27469087	hypothetical protein	21.30%
260	27469100	hypothetical protein	22.50%
261	27469108	hypothetical protein	35.35%
262	27469120	hypothetical protein	27.45%
263	27469121	Mobile element protein	30.36%
264	27469122	ATPase involved in DNA repair	27.04%
265	27469123	hypothetical protein	31.37%
266	27469124	hypothetical protein	30.21%
267	27469142	Glutamate synthase [NADPH] large chain (EC 1.4.1.13)	29.24%
268	27469153	Mobile element protein	32.48%
269	27469155	FIG01109065: hypothetical protein	38.51%
270	27469168	Type I restriction-modification system, specificity subunit S (EC 3.1.21.3)	44.49%
271	27469169	Type I restriction-modification system, specificity subunit S (EC 3.1.21.3)	44.05%
272	27469222	hypothetical protein	47.37%
273	27469249	hypothetical protein	23.93%
274	27469256	hypothetical protein	26.50%
275	27469257	Trp repressor binding protein	31.09%
276	27469258	hypothetical protein	20.51%
277	27469259	FIG01109986: hypothetical protein	29.45%
278	27469260	hypothetical protein within prophage	30.63%
279	27469261	Integrase, superantigen-encoding pathogenicity islands SaPI	33.97%
280	27469262	Integrase, superantigen-encoding pathogenicity islands SaPI	26.10%
281	27469263	Integrase, superantigen-encoding pathogenicity islands SaPI	31.82%
282	27469264	FIG01110068: hypothetical protein	25.28%
283	27469277	hypothetical protein	24.81%
284	27469279	hypothetical protein	21.21%
285	27469281	hypothetical protein	36.90%
286	27469287	hypothetical protein	28.03%
287	27469313	Antiadhesin Pls, binding to squamous nasal epithelial cells	38.47%
288	27469322	hypothetical protein	31.18%
289	27469331	hypothetical protein	24.07%

Highlight rule:

GC content *Staphylococcus epidermidis*
32%
>35 %
<29 %
Genes with altered GC content : 71.20%

Table S5: List of unique genes, their putative function and GC Content (%) of *S. epidermidis* NIHLM023.

Cluster id	NIHLM023	Function	GC content
1	1	DNA repair protein RadC	45.00%
2	2	Predicted cell-wall-anchored protein SasA (LPXTG motif)	44.44%
3	3	Antiadhesin Pls, binding to squamous nasal epithelial cells	37.17%
4	4	hypothetical fig|282458.1.peg.573 homolog	34.37%
5	5	hypothetical protein	28.15%
6	6	hypothetical protein	34.53%
7	7	hypothetical protein	26.03%
8	18	LtrC-like protein	30.40%
9	19	hypothetical protein	24.43%
10	20	DNA topoisomerase III (EC 5.99.1.2)	19.30%
11	21	hypothetical protein	27.60%
12	22	hypothetical protein	26.25%
13	26	FIG01110008: hypothetical protein	23.57%
14	34	Cell division protein FtsH (EC 3.4.24.-)	27.54%
15	79	hypothetical protein	27.98%
16	161	Amino acid permease	35.71%
17	171	Histidinol-phosphatase [alternative form] (EC 3.1.3.15)	35.37%
18	207	FIG01110022: hypothetical protein	31.10%
19	208	FIG01110022: hypothetical protein	30.93%
20	210	Universal stress protein family	29.71%
21	211	Sulfate permease	32.99%
22	212	resolvase	25.78%
23	222	Mobile element protein	34.22%
24	232	hypothetical protein	23.08%
25	234	FIG01109310: hypothetical protein	22.37%
26	236	prophage ps3 protein 14	23.53%
27	237	Hypothetical SAR0369 homolog in superantigen-encoding pathogenicity islands SaPI	32.23%
28	238	hypothetical protein	30.82%
29	239	hypothetical protein	24.88%
30	241	Hypothetical SAV0790 homolog in superantigen-encoding pathogenicity islands SaPI	35.05%
31	243	hypothetical protein	27.36%
32	244	Hypothetical SAV0794 homolog in superantigen-encoding pathogenicity islands SaPI	34.27%
33	252	hypothetical protein	23.33%
34	295	Topoisomerase IV subunit B (EC 5.99.1.-)	35.26%
35	428	L-Proline/Glycine betaine transporter ProP	32.00%
36	441	Arsenic efflux pump protein	31.79%
37	485	DNA repair protein RadA	41.06%
38	551	FIG01108272: hypothetical protein	33.43%
39	590	hypothetical protein	28.07%
40	593	hypothetical protein	19.21%
41	594	hypothetical protein	32.19%
42	595	hypothetical protein	26.95%
43	599	Glucose-6-phosphate 1-dehydrogenase (EC 1.1.1.49)	31.48%
44	603	Putative EsaC protein analog (Listeria type 3)	30.33%
45	610	Lmo0066 homolog within ESAT-6 gene cluster, similarity to ADP-ribosylating toxins	28.55%
46	643	CoA-disulfide reductase (EC 1.8.1.14)	33.33%
47	644	CoA-disulfide reductase (EC 1.8.1.14)	35.93%
48	652	Mobile element protein	29.08%

49	653	Mobile element protein	27.19%
50	664	Na(+) H(+) antiporter subunit A	31.06%
51	699	Transcriptional regulator, HxlR family	29.17%
52	700	hypothetical protein	24.00%
53	701	hypothetical protein	22.76%
54	702	Mobile element protein	34.66%
55	759	ComF operon protein A, DNA transporter ATPase	34.97%
56	780	FIG01278029: hypothetical protein	35.58%
57	801	FIG01110429: hypothetical protein	24.24%
58	802	Hypothetical SAV0789 homolog in superantigen-encoding pathogenicity islands SaPI	31.31%
59	809	hypothetical protein	31.84%
60	812	hypothetical protein	23.98%
61	813	hypothetical protein	22.55%
62	815	hypothetical protein	26.80%
63	816	conserved hypothetical protein	23.97%
64	817	PemK family of DNA-binding proteins	28.60%
65	858	FIG01108504: hypothetical protein	32.52%
66	875	Mobile element protein	22.00%
67	878	acetyltransferase (GNAT) family protein	36.47%
68	967	Phosphinothricin N-acetyltransferase (EC 2.3.1.-)	33.33%
69	968	Phosphinothricin N-acetyltransferase (EC 2.3.1.-)	30.16%
70	1046	Predicted ring-cleavage extradiol dioxygenase	29.89%
71	1047	Predicted ring-cleavage extradiol dioxygenase	29.60%
72	1165	Mobile element protein	26.46%
73	1195	hypothetical protein	31.62%
74	1265	Phenylalanyl-tRNA synthetase domain protein (Bsu YtpR)	31.78%
75	1304	Phosphoenolpyruvate carboxykinase [ATP] (EC 4.1.1.49)	28.33%
76	1337	hypothetical protein	28.57%
77	1348	ATP-binding protein p271	35.00%
78	1349	short chain dehydrogenase	35.45%
79	1350	FIG01119053: hypothetical protein	36.61%
80	1351	Transcriptional regulator, AraC family	29.80%
81	1352	hypothetical protein	25.69%
82	1353	Replication-associated protein	25.14%
83	1355	CDP-glycerol:poly(glycerophosphate) glycerophosphotransferase (EC 2.7.8.12)	29.18%
84	1359	hypothetical protein	28.17%
85	1383	DUF1541 domain-containing protein	34.03%
86	1384	DUF1541 domain-containing protein	35.19%
87	1387	Hypothetical cytosolic protein	27.22%
88	1388	hypothetical protein	25.14%
89	1394	Mobile element protein	29.64%
90	1403	hypothetical protein	25.08%
91	1404	hypothetical protein	27.33%
92	1406	hypothetical protein	27.22%
93	1407	FIG01107856: hypothetical protein	34.36%
94	1409	hypothetical protein	35.42%
95	1410	hypothetical protein	21.01%
96	1415	Transcriptional regulator, DeoR family protein	35.15%
97	1420	Superfamily II DNA/RNA helicases, SNF2 family	28.11%
98	1421	Superfamily II DNA/RNA helicases, SNF2 family	28.79%
99	1422	FIG01252477: hypothetical protein	29.15%
100	1423	Type III restriction-modification system StyLTI enzyme res (EC 3.1.21.5)	27.49%

101	1424	Type III restriction-modification system methylation subunit (EC 2.1.1.72)	27.63%
102	1493	ThiJ/PfpI family	27.75%
103	1497	hypothetical protein	22.76%
104	1498	hypothetical protein	25.66%
105	1529	Hypothetical transmembrane protein coupled to NADH-ubiquinone oxidoreductase chain 5 homolog	36.39%
106	1588	UDP-N-acetylglucosamine--N-acetylmuramyl-(pentapeptide) pyrophosphoryl-undecaprenol N-acetylglucosamine transferase (EC 2.4.1.227)	30.92%
107	1589	UDP-N-acetylglucosamine--N-acetylmuramyl-(pentapeptide) pyrophosphoryl-undecaprenol N-acetylglucosamine transferase (EC 2.4.1.227)	32.41%
108	1691	Mobile element protein	27.15%
109	1695	Glycine dehydrogenase [decarboxylating] (glycine cleavage system P1 protein) (EC 1.4.4.2)	33.12%
110	1785	hypothetical protein	32.46%
111	1859	Repetitive hypothetical protein near ESAT cluster, SA0282 homolog	29.63%
112	1860	Repetitive hypothetical protein near ESAT cluster, SA0282 homolog	29.41%
113	1915	Glutamate synthase [NADPH] large chain (EC 1.4.1.13)	27.99%
114	1934	Protein export cytoplasm protein SecA2 ATPase RNA helicase (TC 3.A.5.1.1)	33.41%
115	1935	Protein export cytoplasm protein SecA2 ATPase RNA helicase (TC 3.A.5.1.1)	33.15%
116	1937	Accessory secretory protein Asp3	33.33%
117	1980	Triacylglycerol lipase (EC 3.1.1.3)	33.13%
118	1995	Fosfomycin resistance protein FosB	26.23%
119	2022	hypothetical protein	17.46%
120	2024	FIG01108182: hypothetical protein	30.07%
121	2025	FIG01108182: hypothetical protein	27.51%
122	2037	FIG01108841: hypothetical protein	30.27%
123	2038	FIG01108606: hypothetical protein	27.89%
124	2058	Mobile element protein	26.46%
125	2059	hypothetical protein	42.22%
126	2062	hypothetical protein	20.00%
127	2080	Peptide chain release factor 3	35.67%
128	2091	hypothetical protein	21.67%
129	2105	Naphthoate synthase (EC 4.1.3.36)	37.25%
130	2113	FIG01110008: hypothetical protein	26.16%
131	2115	CDS_ID OB3289	32.14%
132	2119	Lmo0069 homolog within ESAT-6 gene cluster	32.33%
133	2120	FIG01110734: hypothetical protein	27.34%
134	2121	FIG01110008: hypothetical protein	26.16%
135	2144	Mobile element protein	33.83%
136	2174	FIG01108126: hypothetical protein	29.47%
137	2175	hypothetical protein	23.13%
138	2176	hypothetical protein	33.11%
139	2177	Phage protein	28.70%
140	2178	N-acetylmuramoyl-L-alanine amidase, family 2 (EC 3.5.1.28)	35.19%
141	2179	Bifunctional autolysin Atl / N-acetylmuramoyl-L-alanine amidase (EC 3.5.1.28)/ endo-beta-N-acetylglucosaminidase (EC 3.2.1.96)	30.60%
142	2180	Probable holin	27.27%
143	2181	hypothetical protein	29.89%
144	2182	Phage minor structural protein	28.94%
145	2183	Phage minor structural protein	28.81%
146	2184	Phage minor structural protein	28.60%
147	2185	Phage protein	26.50%
148	2186	Phage tail length tape-measure protein	30.23%
149	2187	Phage protein	27.68%
150	2188	Phage protein	31.01%

151	2189	Phage tail protein	35.45%
152	2190	Phage protein	24.49%
153	2191	Phage protein	28.49%
154	2192	Phage protein	29.37%
155	2193	Phage protein	29.05%
156	2194	Phage major tail protein	30.75%
157	2195	Phage major capsid protein	32.44%
158	2196	FIG01109296: hypothetical protein	28.76%
159	2198	FIG01107943: hypothetical protein	25.31%
160	2200	Phage portal protein	29.12%
161	2201	Phage terminase, large subunit	28.86%
162	2202	Phage terminase, small subunit	30.54%
163	2203	hypothetical protein	22.22%
164	2204	prophage LambdaBa01, positive control factor Xpf	25.66%
165	2205	hypothetical protein	18.99%
166	2206	Dimeric dUTPase (EC 3.6.1.23)	30.73%
167	2207	Phage protein	29.67%
168	2208	hypothetical protein	31.67%
169	2209	hypothetical protein	31.58%
170	2210	Phage replication initiation protein	31.12%
171	2211	Single stranded DNA-binding protein, phage-associated	29.26%
172	2212	Hypothetical protein, phi-ETA orf17 homolog [SA bacteriophages 11, Mu50B]	30.60%
173	2213	FIG01109301: hypothetical protein	27.59%
174	2214	Phage protein	26.22%
175	2215	hypothetical protein	28.72%
176	2216	hypothetical protein	27.96%
177	2217	Phage protein	29.68%
178	2218	Phage repressor	27.30%
179	2219	hypothetical protein	24.73%
180	2295	Signal recognition particle receptor protein FtsY (=alpha subunit) (TC 3.A.5.1.1)	35.88%
181	2320	Mobile element protein	36.32%

Highlight rule:

GC content *Staphylococcus epidermidis*
32%
>35 %
<29 %
Genes with altered GC content : 59.0%

Table S6: List of unique genes, their putative function and GC Content (%) of *S. epidermidis* SE2.9.

Cluster id	SE2.9	Function	GC content
1	33	hypothetical protein transposon-related	27.27%
2	34	hypothetical protein	25.04%
3	273	Mobile element protein	29.23%
4	306	hypothetical protein	33.33%
5	636	Cell wall surface anchor family protein	41.44%
6	637	hypothetical protein	41.57%
7	638	Cell wall surface anchor family protein	34.73%
8	656	FIG01107925: hypothetical protein	33.33%
9	731	recombination protein	26.24%
10	755	hypothetical protein	26.94%
11	756	Mobile element protein	28.16%
12	758	Transcriptional regulator, Cro/CI family	27.54%
13	759	Inner membrane protein translocase component YidC, short form OxaI-like	30.58%
14	760	Transcriptional regulator, Cro/CI family	24.66%
15	761	hypothetical protein	28.96%
16	762	hypothetical protein	26.67%
17	763	FIG01108994: hypothetical protein	26.60%
18	764	ATP-binding protein p271	32.35%
19	766	ABC transporter ATP-binding protein uup	30.43%
20	773	hypothetical protein	33.33%
21	775	Transcriptional regulator, XRE family	27.27%
22	776	hypothetical protein	25.16%
23	835	FIG01110022: hypothetical protein	31.42%
24	841	hypothetical protein	30.89%
25	851	Dihydroxy-acid dehydratase (EC 4.2.1.9)	39.74%
26	876	hypothetical protein	25.93%
27	877	Hypothetical SAV0787 homolog in superantigen-encoding pathogenicity islands SaPI	32.71%
28	878	hypothetical protein	28.47%
29	879	Hypothetical SAV0788 homolog in superantigen-encoding pathogenicity islands SaPI	30.00%
30	880	FIG01110429: hypothetical protein	28.95%
31	881	Phage protein	29.93%
32	882	Phage replication protein # ACLAME 208	33.83%
33	883	Phage protein	27.84%
34	885	hypothetical protein	31.46%
35	886	Mobile element protein	27.61%
36	887	Mobile element protein	25.26%
37	888	Mobile element protein	28.48%
38	889	Mobile element protein	34.46%
39	890	Phage replication initiation protein	26.33%
40	891	Mobile element protein	34.07%
41	892	hypothetical protein	30.65%
42	893	Hypothetical SAV0796 homolog in superantigen-encoding pathogenicity islands SaPI	31.97%
43	894	FIG01110123: hypothetical protein	29.17%
44	895	Putative terminase, superantigen-encoding pathogenicity islands SaPI	37.87%
45	897	hypothetical protein	28.13%
46	902	hypothetical protein	24.15%
47	904	hypothetical protein	34.01%
48	906	hypothetical protein	31.62%

49	907	hypothetical protein	32.82%
50	909	hypothetical protein	24.56%
51	910	Mobile element protein	33.93%
52	911	hypothetical protein	25.40%
53	912	hypothetical protein	20.54%
54	921	Glucose-6-phosphate 1-dehydrogenase (EC 1.1.1.49)	27.59%
55	922	hypothetical protein	29.82%
56	926	hypothetical protein	28.92%
57	927	CDS_ID OB3289	38.10%
58	933	hypothetical protein	27.16%
59	937	Hypothetical protein, CF-38 family	26.97%
60	938	Hypothetical protein, CF-38 family	28.57%
61	1058	hypothetical protein	30.89%
62	1063	hypothetical protein	31.84%
63	1066	"Putative terminase, superantigen-encoding pathogenicity islands SaPI"	35.14%
64	1067	Integrase, superantigen-encoding pathogenicity islands SaPI	35.04%
65	1068	Integrase, superantigen-encoding pathogenicity islands SaPI	32.58%
66	1069	hypothetical protein	21.30%
67	1071	"Transcriptional regulator, AraC family"	27.02%
68	1072	fibronectin-binding protein	25.23%
69	1073	hypothetical protein	25.16%
70	1075	Phage protein	30.70%
71	1076	hypothetical protein	38.10%
72	1077	hypothetical protein	30.64%
73	1078	hypothetical protein	26.83%
74	1079	hypothetical protein	28.44%
75	1080	hypothetical protein	26.81%
76	1081	hypothetical protein	35.14%
77	1082	site-specific recombinase, resolvase family	32.54%
78	1083	hypothetical protein	26.67%
79	1084	Mobile element protein	34.07%
80	1089	FIG01108272: hypothetical protein	33.38%
81	1165	Hypothetical SAV0798 homolog in superantigen-encoding pathogenicity islands SaPI	32.55%
82	1166	Phage protein	29.53%
83	1168	hypothetical protein	24.69%
84	1169	hypothetical protein	23.64%
85	1170	Transcriptional regulator, AraC family	28.32%
86	1171	hypothetical protein	22.92%
87	1178	Mobile element protein	33.79%
88	1179	Replication protein	29.46%
89	1180	hypothetical protein	27.45%
90	1181	hypothetical protein	41.03%
91	1182	hypothetical protein	30.71%
92	1183	hypothetical protein	24.85%
93	1186	Putative EsaC protein analog (Listeria type 3)	30.42%
94	1188	Mobile element protein	28.23%
95	1189	Mobile element protein	33.92%
96	1219	Extracellular protein	30.98%
97	1221	hypothetical protein	29.63%
98	1222	Type I restriction-modification system, restriction subunit R (EC 3.1.21.3)	30.85%
99	1223	FIG01109984: hypothetical protein	28.46%
100	1224	"Glycosyl transferase, group 2 family protein"	31.75%

101	1225	FIG01109073: hypothetical protein	33.45%
102	1226	cell surface protein precursor	34.34%
103	1227	4-hydroxythreonine-4-phosphate dehydrogenase (EC 1.1.1.262)	30.00%
104	1228	Hydrolase, HAD subfamily IIIA	27.47%
105	1229	hypothetical protein	30.41%
106	1231	hypothetical protein	28.63%
107	1232	hypothetical protein	37.46%
108	1233	hypothetical protein	30.01%
109	1234	DNA repair protein RadC	34.00%
110	1238	hypothetical protein	33.33%
111	1239	putative primase	31.75%
112	1240	FIG01108642: hypothetical protein	34.14%
113	1241	FIG01109227: hypothetical protein	35.15%
114	1242	hypothetical protein	26.63%
115	1243	FIG00629270: hypothetical protein	29.05%
116	1244	hypothetical protein	26.14%
117	1269	Ribonuclease P protein component (EC 3.1.26.5)	30.50%
118	1337	hypothetical protein	23.91%
119	1340	OriT nickase Nes	25.73%
120	1341	hypothetical protein	36.31%
121	1342	hypothetical protein	31.17%
122	1459	hypothetical protein	22.67%
123	1461	hypothetical protein	24.77%
124	1471	Hypothetical SAV0794 homolog in superantigen-encoding pathogenicity islands SaPI	33.64%
125	1493	Transcriptional regulator of biofilm formation (AraC/XylS family)	28.87%
126	1559	Mobile element protein	29.50%
127	1593	Arsenic efflux pump protein	32.36%
128	1667	metal-dependent phosphohydrolase	31.82%
129	1928	hypothetical protein	26.15%
130	1929	hypothetical protein	24.04%
131	1930	hypothetical protein	22.77%
132	2000	hypothetical protein	29.27%
133	2003	Multidrug resistance protein	36.81%
134	2027	hypothetical protein	30.39%
135	2208	Ferrous iron transport protein B	32.02%
136	2209	Ferrous iron transport protein B	34.47%
137	2250	hypothetical protein	30.07%
138	2270	hypothetical protein	24.03%
139	2296	FIG01109350: hypothetical protein	26.56%
140	2332	FIG01109350: hypothetical protein	28.47%
141	2341	Hypothetical SAV0801 homolog in superantigen-encoding pathogenicity islands SaPI	25.28%
142	2342	hypothetical protein	17.09%

Highlight rule:

GC content *Staphylococcus epidermidis*
32%
>35 %
<29 %
Genes with altered GC content : 54.0%

Table S7: List of unique genes, their putative function and GC Content (%) of *S. epidermidis* CIM40.

Cluster id	NIHLM023	Function	GC content
1	1	DNA repair protein RadC	45.00%
2	2	Predicted cell-wall-anchored protein SasA (LPXTG motif)	44.44%
3	3	Antiadhesin Pls, binding to squamous nasal epithelial cells	37.17%
4	4	hypothetical fig|282458.1.peg.573 homolog	34.37%
5	5	hypothetical protein	28.15%
6	6	hypothetical protein	34.53%
7	7	hypothetical protein	26.03%
8	18	LtrC-like protein	30.40%
9	19	hypothetical protein	24.43%
10	20	DNA topoisomerase III (EC 5.99.1.2)	19.30%
11	21	hypothetical protein	27.60%
12	22	hypothetical protein	26.25%
13	26	FIG01110008: hypothetical protein	23.57%
14	34	Cell division protein FtsH (EC 3.4.24.-)	27.54%
15	79	hypothetical protein	27.98%
16	161	Amino acid permease	35.71%
17	171	Histidinol-phosphatase [alternative form] (EC 3.1.3.15)	35.37%
18	207	FIG01110022: hypothetical protein	31.10%
19	208	FIG01110022: hypothetical protein	30.93%
20	210	Universal stress protein family	29.71%
21	211	Sulfate permease	32.99%
22	212	resolvase	25.78%
23	222	Mobile element protein	34.22%
24	232	hypothetical protein	23.08%
25	234	FIG01109310: hypothetical protein	22.37%
26	236	prophage ps3 protein 14	23.53%
27	237	Hypothetical SAR0369 homolog in superantigen-encoding pathogenicity islands SaPI	32.23%
28	238	hypothetical protein	30.82%
29	239	hypothetical protein	24.88%
30	241	Hypothetical SAV0790 homolog in superantigen-encoding pathogenicity islands SaPI	35.05%
31	243	hypothetical protein	27.36%
32	244	Hypothetical SAV0794 homolog in superantigen-encoding pathogenicity islands SaPI	34.27%
33	252	hypothetical protein	23.33%
34	295	Topoisomerase IV subunit B (EC 5.99.1.-)	35.26%
35	428	L-Proline/Glycine betaine transporter ProP	32.00%
36	441	Arsenic efflux pump protein	31.79%
37	485	DNA repair protein RadA	41.06%
38	551	FIG01108272: hypothetical protein	33.43%
39	590	hypothetical protein	28.07%
40	593	hypothetical protein	19.21%
41	594	hypothetical protein	32.19%
42	595	hypothetical protein	26.95%
43	599	Glucose-6-phosphate 1-dehydrogenase (EC 1.1.1.49)	31.48%
44	603	Putative EsaC protein analog (Listeria type 3)	30.33%
45	610	Lmo0066 homolog within ESAT-6 gene cluster, similarity to ADP-ribosylating toxins	28.55%
46	643	CoA-disulfide reductase (EC 1.8.1.14)	33.33%
47	644	CoA-disulfide reductase (EC 1.8.1.14)	35.93%
48	652	Mobile element protein	29.08%

49	653	Mobile element protein	27.19%
50	664	Na(+) H(+) antiporter subunit A	31.06%
51	699	Transcriptional regulator, HxlR family	29.17%
52	700	hypothetical protein	24.00%
53	701	hypothetical protein	22.76%
54	702	Mobile element protein	34.66%
55	759	ComF operon protein A, DNA transporter ATPase	34.97%
56	780	FIG01278029: hypothetical protein	35.58%
57	801	FIG01110429: hypothetical protein	24.24%
58	802	Hypothetical SAV0789 homolog in superantigen-encoding pathogenicity islands SaPI	31.31%
59	809	hypothetical protein	31.84%
60	812	hypothetical protein	23.98%
61	813	hypothetical protein	22.55%
62	815	hypothetical protein	26.80%
63	816	conserved hypothetical protein	23.97%
64	817	PemK family of DNA-binding proteins	28.60%
65	858	FIG01108504: hypothetical protein	32.52%
66	875	Mobile element protein	22.00%
67	878	acetyltransferase (GNAT) family protein	36.47%
68	967	Phosphinothricin N-acetyltransferase (EC 2.3.1.-)	33.33%
69	968	Phosphinothricin N-acetyltransferase (EC 2.3.1.-)	30.16%
70	1046	Predicted ring-cleavage extradiol dioxygenase	29.89%
71	1047	Predicted ring-cleavage extradiol dioxygenase	29.60%
72	1165	Mobile element protein	26.46%
73	1195	hypothetical protein	31.62%
74	1265	Phenylalanyl-tRNA synthetase domain protein (Bsu YtpR)	31.78%
75	1304	Phosphoenolpyruvate carboxykinase [ATP] (EC 4.1.1.49)	28.33%
76	1337	hypothetical protein	28.57%
77	1348	ATP-binding protein p271	35.00%
78	1349	short chain dehydrogenase	35.45%
79	1350	FIG01119053: hypothetical protein	36.61%
80	1351	Transcriptional regulator, AraC family	29.80%
81	1352	hypothetical protein	25.69%
82	1353	Replication-associated protein	25.14%
83	1355	CDP-glycerol:poly(glycerophosphate) glycerophosphotransferase (EC 2.7.8.12)	29.18%
84	1359	hypothetical protein	28.17%
85	1383	DUF1541 domain-containing protein	34.03%
86	1384	DUF1541 domain-containing protein	35.19%
87	1387	Hypothetical cytosolic protein	27.22%
88	1388	hypothetical protein	25.14%
89	1394	Mobile element protein	29.64%
90	1403	hypothetical protein	25.08%
91	1404	hypothetical protein	27.33%
92	1406	hypothetical protein	27.22%
93	1407	FIG01107856: hypothetical protein	34.36%
94	1409	hypothetical protein	35.42%
95	1410	hypothetical protein	21.01%
96	1415	Transcriptional regulator, DeoR family protein	35.15%
97	1420	Superfamily II DNA/RNA helicases, SNF2 family	28.11%
98	1421	Superfamily II DNA/RNA helicases, SNF2 family	28.79%
99	1422	FIG01252477: hypothetical protein	29.15%
100	1423	Type III restriction-modification system StyLTI enzyme res (EC 3.1.21.5)	27.49%

101	1424	Type III restriction-modification system methylation subunit (EC 2.1.1.72)	27.63%
102	1493	ThiJ/PfpI family	27.75%
103	1497	hypothetical protein	22.76%
104	1498	hypothetical protein	25.66%
105	1529	Hypothetical transmembrane protein coupled to NADH-ubiquinone oxidoreductase chain 5 homolog	36.39%
106	1588	UDP-N-acetylglucosamine--N-acetylmuramyl-(pentapeptide) pyrophosphoryl-undecaprenol N-acetylglucosamine transferase (EC 2.4.1.227)	30.92%
107	1589	UDP-N-acetylglucosamine--N-acetylmuramyl-(pentapeptide) pyrophosphoryl-undecaprenol N-acetylglucosamine transferase (EC 2.4.1.227)	32.41%
108	1691	Mobile element protein	27.15%
109	1695	Glycine dehydrogenase [decarboxylating] (glycine cleavage system P1 protein) (EC 1.4.4.2)	33.12%
110	1785	hypothetical protein	32.46%
111	1859	Repetitive hypothetical protein near ESAT cluster, SA0282 homolog	29.63%
112	1860	Repetitive hypothetical protein near ESAT cluster, SA0282 homolog	29.41%
113	1915	Glutamate synthase [NADPH] large chain (EC 1.4.1.13)	27.99%
114	1934	Protein export cytoplasm protein SecA2 ATPase RNA helicase (TC 3.A.5.1.1)	33.41%
115	1935	Protein export cytoplasm protein SecA2 ATPase RNA helicase (TC 3.A.5.1.1)	33.15%
116	1937	Accessory secretory protein Asp3	33.33%
117	1980	Triacylglycerol lipase (EC 3.1.1.3)	33.13%
118	1995	Fosfomycin resistance protein FosB	26.23%
119	2022	hypothetical protein	17.46%
120	2024	FIG01108182: hypothetical protein	30.07%
121	2025	FIG01108182: hypothetical protein	27.51%
122	2037	FIG01108841: hypothetical protein	30.27%
123	2038	FIG01108606: hypothetical protein	27.89%
124	2058	Mobile element protein	26.46%
125	2059	hypothetical protein	42.22%
126	2062	hypothetical protein	20.00%
127	2080	Peptide chain release factor 3	35.67%
128	2091	hypothetical protein	21.67%
129	2105	Naphthoate synthase (EC 4.1.3.36)	37.25%
130	2113	FIG01110008: hypothetical protein	26.16%
131	2115	CDS_ID OB3289	32.14%
132	2119	Lmo0069 homolog within ESAT-6 gene cluster	32.33%
133	2120	FIG01110734: hypothetical protein	27.34%
134	2121	FIG01110008: hypothetical protein	26.16%
135	2144	Mobile element protein	33.83%
136	2174	FIG01108126: hypothetical protein	29.47%
137	2175	hypothetical protein	23.13%
138	2176	hypothetical protein	33.11%
139	2177	Phage protein	28.70%
140	2178	N-acetylmuramoyl-L-alanine amidase, family 2 (EC 3.5.1.28)	35.19%
141	2179	Bifunctional autolysin Atl / N-acetylmuramoyl-L-alanine amidase (EC 3.5.1.28)/ endo-beta-N-acetylglucosaminidase (EC 3.2.1.96)	30.60%
142	2180	Probable holin	27.27%
143	2181	hypothetical protein	29.89%
144	2182	Phage minor structural protein	28.94%
145	2183	Phage minor structural protein	28.81%
146	2184	Phage minor structural protein	28.60%
147	2185	Phage protein	26.50%
148	2186	Phage tail length tape-measure protein	30.23%
149	2187	Phage protein	27.68%
150	2188	Phage protein	31.01%
151	2189	Phage tail protein	35.45%
152	2190	Phage protein	24.49%

153	2191	Phage protein	28.49%
154	2192	Phage protein	29.37%
155	2193	Phage protein	29.05%
156	2194	Phage major tail protein	30.75%
157	2195	Phage major capsid protein	32.44%
158	2196	FIG01109296: hypothetical protein	28.76%
159	2198	FIG01107943: hypothetical protein	25.31%
160	2200	Phage portal protein	29.12%
161	2201	Phage terminase, large subunit	28.86%
162	2202	Phage terminase, small subunit	30.54%
163	2203	hypothetical protein	22.22%
164	2204	prophage LambdaBa01, positive control factor Xpf	25.66%
165	2205	hypothetical protein	18.99%
166	2206	Dimeric dUTPase (EC 3.6.1.23)	30.73%
167	2207	Phage protein	29.67%
168	2208	hypothetical protein	31.67%
169	2209	hypothetical protein	31.58%
170	2210	Phage replication initiation protein	31.12%
171	2211	Single stranded DNA-binding protein, phage-associated	29.26%
172	2212	Hypothetical protein, phi-ETA orf17 homolog [SA bacteriophages 11, Mu50B]	30.60%
173	2213	FIG01109301: hypothetical protein	27.59%
174	2214	Phage protein	26.22%
175	2215	hypothetical protein	28.72%
176	2216	hypothetical protein	27.96%
177	2217	Phage protein	29.68%
178	2218	Phage repressor	27.30%
179	2219	hypothetical protein	24.73%
180	2295	Signal recognition particle receptor protein FtsY (=alpha subunit) (TC 3.A.5.1.1)	35.88%
181	2320	Mobile element protein	36.32%

Highlight rule:

GC content *Staphylococcus epidermidis*
32%
>35 %
<29 %
Genes with altered GC content : 63.0%

Table S8: COG classification of unique genes in *S. epidermidis* ATCC12228, NIHLM023, SE2.9 and CIM40.

	ATCC12228		NIHLM023		SE2.9		CIM40	
**#COG class**	**description**	**count**	**description**	**count**	**description**	**count**	**description**	**count**
**C**	Energy production and conversion	7	Energy production and conversion	1	Energy production and conversion	0	Energy production and conversion	0
**D**	Cell cycle control, cell division, chromosome partitioning	1	Cell cycle control, cell division, chromosome partitioning	1	Cell cycle control, cell division, chromosome partitioning	0	Cell cycle control, cell division, chromosome partitioning	2
**E**	Amino acid transport and metabolism	5	Amino acid transport and metabolism	3	Amino acid transport and metabolism	2	Amino acid transport and metabolism	1
**F**	Nucleotide transport and metabolism	0	Nucleotide transport and metabolism	0	Nucleotide transport and metabolism	0	Nucleotide transport and metabolism	2
**G**	Carbohydrate transport and metabolism	1	Carbohydrate transport and metabolism	3	Carbohydrate transport and metabolism	4	Carbohydrate transport and metabolism	4
**H**	Coenzyme transport and metabolism	0	Coenzyme transport and metabolism	1	Coenzyme transport and metabolism	0	Coenzyme transport and metabolism	2
**I**	Lipid transport and metabolism	2	Lipid transport and metabolism	0	Lipid transport and metabolism	0	Lipid transport and metabolism	1
**J**	Translation, ribosomal structure and biogenesis	3	Translation, ribosomal structure and biogenesis	2	Translation, ribosomal structure and biogenesis	0	Translation, ribosomal structure and biogenesis	0
**K**	Transcription	5	Transcription	6	Transcription	5	Transcription	4
**L**	Replication, recombination and repair	13	Replication, recombination and repair	14	Replication, recombination and repair	15	Replication, recombination and repair	4
**M**	Cell wall/membrane/envelope biogenesis	6	Cell wall/membrane/envelope biogenesis	6	Cell wall/membrane/envelope biogenesis	1	Cell wall/membrane/envelope biogenesis	4
**N**	Cell motility	0	Cell motility	1	Cell motility	0	Cell motility	0
**O**	Posttranslational modification, protein turnover, chaperones	2	Posttranslational modification, protein turnover, chaperones	2	Posttranslational modification, protein turnover, chaperones	0	Posttranslational modification, protein turnover, chaperones	1
**P**	Inorganic ion transport and metabolism	7	Inorganic ion transport and metabolism	3	Inorganic ion transport and metabolism	3	Inorganic ion transport and metabolism	6
**Q**	Secondary metabolites biosynthesis, transport and catabolism	1	Secondary metabolites biosynthesis, transport and catabolism	0	Secondary metabolites biosynthesis, transport and catabolism	0	Secondary metabolites biosynthesis, transport and catabolism	2
**R**	General function prediction only	10	General function prediction only	10	General function prediction only	7	General function prediction only	12
**S**	Function unknown	5	Function unknown	5	Function unknown	10	Function unknown	5
**T**	Signal transduction mechanisms	7	Signal transduction mechanisms	2	Signal transduction mechanisms	1	Signal transduction mechanisms	2
**U**	Intracellular trafficking, secretion, and vesicular transport	1	Intracellular trafficking, secretion, and vesicular transport	3	Intracellular trafficking, secretion, and vesicular transport	1	Intracellular trafficking, secretion, and vesicular transport	1
**V**	Defense mechanisms	6	Defense mechanisms	1	Defense mechanisms	4	Defense mechanisms	3
**None**	No COG category	207	No COG category	117	No COG category	89	No COG category	141

Table S9: List of unique genes in RESE annotated in hyper-variable regions generated by BRIG, their putative function and GC Content (%).

Region	ORF no.	start	stop	strand	function	GC content (%)
**RESE-GI-1**	**1**	324	1	-	putative lipoprotein	27.16
	**2**	553	1116	+	Type I restriction-modification system, restriction subunit R (EC 3.1.21.3)	30.85
	**3**	1207	2274	+	intercellular adhesion protein C	28.46
	**4**	2289	4184	+	Glycosyl transferase, group 2 family protein	31.75
	**5**	5490	4654	-	serine protease family protein	33.45
	**6**	6697	5510	-	cell surface protein precursor	34.34
	**7**	7436	6777	-	4-hydroxythreonine-4-phosphate dehydrogenase (EC 1.1.1.262) (pdxA)	30
	**8**	7578	8942	+	Hydrolase, HAD subfamily IIIA, vanomycin resistance protein VanB	27.47
	**9**	9139	9291	-	putative peptide-methionine (S)-S-oxide reductase (msrA3)	35.29
	**10**	10703	10260	-	LPXTG protein-like protein	30.4
	**11**	11292	10765	-	hypothetical protein	31.25
	**12**	11915	11427	-	hypothetical protein	28.62
	**13**	12221	11931	-	putative membrane protein	37.45
	**14**	13145	12273	-	transcriptional regulator	30.01
	**15**	13761	13315	-	DNA repair protein RadC	34
	**16**	14295	13789	-	conserved hypothetical protein	33.53
	**17**	14622	14311	-	putative uncharacterized protein	32.37
	**18**	15059	14718	-	conserved hypothetical protein	37.42
	**19**	16838	15162	-	Cassette chromosome recombinase B	33.69
	**20**	17111	16995	-	hypothetical protein	33.33
	**21**	18708	17065	-	putative primase	31.75
	**22**	19076	18705	-	conserved hypothetical protein	34.13
	**23**	20169	19069	-	DNA polymerase family A	35.14
	**24**	21872	20397	-	hypothetical protein	26.62
	**25**	22821	21958	-	endonuclease	29.05
	**26**	23324	23019	-	HNH endonuclease	26.14
	**27**	23911	23570	-	50S rRNA methyltransferase	38.88
**RESE-GI-2**	**1**	1395	258	-	hypothetical protein	23.91
	**2**	1059	535	-	DNA-binding protein	20.95
	**3**	1857	1183	-	Mobile element protein	34.22
**RESE-GI-3**	**1**	1	202	-	general stress protein	30.34
	**2**	393	743	+	conserved hypothetical protein	29.05
	**3**	6419	1212	-	putative dnd system-associated protein 2	26.15
	**4**	7755	6400	-	putative dnd system-associated protein 1	24.04
	**5**	9650	7758	-	putative dnd system-associated protein 3	22.76
**RESE-GI-4**	**1**	1390	125	-	fic/DOC family protein	25.27
	**2**	1739	1542	-	XRE family transcriptional regulator	27.27
	**3**	2713	2910	+	Integrase	29.29
**RESE-GI-5**	**1**	212	397	-	integrase	34.4
	**2**	499	1380	+	conserved domain protein	35.39
	**3**	1384	2002	+	hypothetical protein	21.03
